# Expectations and Prior Experiences Associated With Adverse Effects of COVID-19 Vaccination

**DOI:** 10.1001/jamanetworkopen.2023.4732

**Published:** 2023-03-27

**Authors:** Ingmar Schäfer, Jan Hendrik Oltrogge, Yvonne Nestoriuc, Claire V. Warren, Stefanie Brassen, Maximilian Blattner, Dagmar Lühmann, Alexandra Tinnermann, Martin Scherer, Christian Büchel

**Affiliations:** 1 Institute and Outpatients Clinic of General Practice/Primary Care, University Medical Center Hamburg-Eppendorf, Hamburg, Germany; 2 Clinical Psychology, Helmut-Schmidt-University/University of the Federal Armed Forces Hamburg, Hamburg, Germany; 3 Institute of Systems Neuroscience, University Medical Center Hamburg-Eppendorf, Hamburg, Germany

## Abstract

**Importance:**

Uptake of vaccination against COVID-19 is strongly affected by concerns about adverse effects. Research on nocebo effects suggests that these concerns can amplify symptom burden.

**Objective:**

To investigate whether positive and negative expectations prior to COVID-19 vaccination are associated with systemic adverse effects.

**Design, Setting, and Participants:**

This prospective cohort study analyzed the association of expected benefits and risks of vaccination, adverse effects at first vaccination, and observed adverse effects in close contacts with severity of systemic adverse effects among adults receiving a second dose of messenger RNA (mRNA)–based vaccines between August 16 and 28, 2021. A total of 7771 individuals receiving the second dose at a state vaccination center in Hamburg, Germany, were invited to participate; of these, 5370 did not respond, 535 provided incomplete information, and 188 were excluded retrospectively. The mobile application m-Path was used for data collection.

**Main Outcomes and Measures:**

Primary outcome was a composite severity index of systemic adverse effects in 12 symptom areas measured once daily with an electronic symptom diary over 7 consecutive days. Data were analyzed by mixed-effects multivariable ordered logistic regression adjusted for prevaccine symptom levels and observation times.

**Results:**

A total of 10 447 observations from 1678 individuals receiving vaccinations (BNT162b2 [Pfizer BioNTech] in 1297 [77.3%] and mRNA-1273 [Moderna] in 381 [22.7%]) were collected. The participants’ median age was 34 (IQR, 27-44) years, and 862 (51.4%) were women. The risk for more severe adverse effects was higher for persons expecting a lower benefit of vaccination (odds ratio [OR] for higher expectations, 0.72 [95% CI, 0.63-0.83]; *P* < .001), expecting higher adverse effects of vaccination (OR, 1.39 [95% CI, 1.23-1.58]; *P* < .001), having experienced higher symptom burden at the first vaccination (OR, 1.60 [95% CI, 1.42-1.82]; *P* < .001), scoring higher on the Somatosensory Amplification Scale (OR, 1.21 [95% CI, 1.06-1.38]; *P* = .004), and if the vaccine mRNA-1273 was given rather than BNT162b2 (OR, 2.45 [95% CI, 2.01-2.99]; *P* < .001). No associations were seen for observed experiences.

**Conclusions and Relevance:**

In this cohort study, several nocebo effects occurred in the first week after COVID-19 vaccination. The severity of systemic adverse effects was associated not only with vaccine-specific reactogenicity but also more negative prior experiences with adverse effects from the first COVID-19 vaccination, more negative expectations regarding vaccination, and tendency to catastrophize instead of normalize benign bodily sensations. Clinician-patient interactions and public vaccine campaigns may both benefit from these insights by optimizing and contextualizing information provided about COVID-19 vaccines.

## Key Points

**Question:**

Are positive and negative expectations prior to COVID-19 vaccination associated with systemic adverse effects in individuals receiving their second dose of COVID-19 vaccines?

**Findings:**

In this cohort study of 1678 participants, expectations of low benefit and high adverse effects, the tendency to catastrophize instead of normalize benign bodily sensations, and prior negative experiences were associated with vaccination adverse effects.

**Meaning:**

These finding suggest that risk factors for nocebo effects exist that can be assessed prior to vaccination and in some cases (ie, negative expectations) can potentially be changed via expectation management strategies.

## Teaser

This cohort study investigates whether positive and negative expectations are associated with systemic adverse effects among adults receiving their second dose of COVID-19 vaccines.

## Introduction

COVID-19 vaccines effectively reduce mortality and disease burden.^1^ Their impact on the ongoing SARS-CoV-2 pandemic, however, depends on effective dissemination and high acceptance in the population. Despite ample vaccine availability, vaccine uptake remains less than desirable in many industrialized countries.^2^ Concerns about potential adverse effects of vaccination are considered an important contributor to the continuing problem of vaccine hesitancy.^3,4,5^

Adverse effects following COVID-19 vaccination include systemic reactions such as myalgia and fever.^6,7^ Psychological factors can contribute to a large extent to symptom burden.^8,9,10^ Nocebo effects are phenomena whereby people taking inert substances experience adverse effects. However, they also influence clinical outcomes in active treatments.^11,12^ For example, in COVID-19 vaccine trials, adverse effects in placebo groups overlapped by 76% (first dose) and 52% (second dose) with those in vaccine groups, while underlying mechanisms were not investigated.^12^

Negative expectations and contextual factors like prior experiences and observed experiences of significant others can amplify nocebo effects.^13^ Nevertheless, participants’ expectations about benefits and risks of vaccines are typically not reported in COVID-19 vaccination trials. Psychological characteristics including anxiety, depression, and the tendency to amplify benign bodily sensations have been associated with higher reported adverse effects for active drugs and nocebo effects.^14,15^ Moreover, positive vaccination attitudes and low concern over COVID-19 vaccine adverse effects were associated with intentions for booster vaccination.^16^ More knowledge on these factors could aid in strategies to attenuate nocebo-related adverse effects.

Therefore, we aimed to investigate whether expectations are associated with systemic adverse effects in individuals receiving their second dose of COVID-19 vaccines. We hypothesized that severity of systemic adverse effects would be increased in participants with lower benefit expectations, with higher expectations of adverse effects, and with more of their personal and observed prior experiences regarding adverse reactions to COVID-19 vaccines, after controlling for prevaccine symptom levels. Furthermore, we hypothesized that higher levels of anxiety and depression as well as a higher tendency to amplify bodily sensations would be associated with increased adverse effect reports.

## Methods

### Study Design, Setting, and Participants

We conducted a longitudinal cohort study at the COVID-19 vaccination center in Hamburg, Germany, which operated from Mondays through Saturdays from 8 am to 8 pm. Study participants were recruited locally between August 16 and 28, 2021. All individuals receiving a second dose of BNT162b2 (Pfizer-BioNTech) or messenger RNA (mRNA)–1273 (Moderna) were eligible for the study if they were 18 years or older, had sufficient German language skills, and had capacity to consent. After vaccination, individuals had to rest for 15 minutes before receiving their vaccination certificate and leaving the center. The project staff handed out a short information leaflet inviting individuals to participate in the study and answered questions. Due to time constraints for recruiting, individuals not fulfilling criteria for eligibility were excluded retrospectively. The study was approved by the local Psychological Ethics Committee at the Center for Psychosocial Medicine of the University Medical Center Hamburg-Eppendorf and followed the Strengthening the Reporting of Observational Studies in Epidemiology (STROBE) reporting guideline. The study leaflet included a QR code facilitating installation of the application m-Path (KU Leuven) developed for clinical assessments and blended care interventions. After informed consent was obtained within the application, participants were asked to complete the baseline questionnaire in the waiting session directly following vaccination. The first follow-up was activated at 9 pm on the same day. The remaining follow-ups were activated at 6 pm on days 2 to 7. A notification was sent when a questionnaire was activated and another reminder followed after 60 minutes. All questionnaires remained available until midnight and expired afterward. Expiration of a questionnaire did not exclude participants from participation, and notifications for future follow-up questionnaires continued as scheduled.

### Outcome Variables

The primary outcome was a composite severity index of systemic adverse effects measured with an electronic symptom diary over 7 consecutive days. Systemic adverse effects of vaccines are defined as whole-body reactions (eg, body aches, fever) occurring in the days following vaccination as opposed to allergic reactions that followed minutes after the injections or local adverse effects that followed within a few hours after the vaccination.^17,18^ The items of the symptom diary were derived from phase 3 trials of BNT162b2^7^ and mRNA-1273^6^ and included aching limbs, chills, deep leg pain, diarrhea, fever, hematomas and/or punctiform hemorrhages, headache, heart pain, joint pain, shortness of breath, tiredness or fatigue, and vomiting (eBoxes 1 and 2 and eMethods in Supplement 1).

At baseline, study participants were instructed to rate the highest severity of these symptom areas during the last 2 weeks before vaccination. At each follow-up, participants were asked to report the current severity in each symptom area. For our multivariable analyses, a composite index of symptom severity was calculated. It was defined by the highest severity in the 12 symptom areas. For example, if a study participant had headache with light severity and tiredness with moderate severity, the composite index showed moderate severity.

### Independent Variables

Independent variables measured expected benefits and risks as well as personal and observed prior experiences related to COVID-19 vaccination and were reported at baseline using a self-developed scale based on an existing questionnaire.^19^ The study participants used a numerical rating scale ranging from “no benefit” to “very high benefit” to rate expected benefit of vaccination. Numerical rating scales ranging from “no risk” to “very high risk” were used to report expected risks connected with COVID-19 infection and vaccination. The 4-item version of the Patient Health Questionnaire was used to screen for anxiety and depression.^20,21^ The Somatosensory Amplification Scale was used to measure the tendency to catastrophize instead of normalize benign bodily sensations such as itching or pain due to insect bites or hunger contraction of stomach.^22,23^

Potential confounders were prespecified based on our experience from other studies and without statistical exploration. They included the vaccine used on the day of recruitment, health status, and sociodemographic data assessed at baseline. Sociodemographic data included age, sex, living arrangements, educational level, and migration status. Educational level was operationalized by highest general and vocational qualification and coded pursuant to the Comparative Analysis of Social Mobility in Industrial Nations classification.^24^ Migration status was assessed by country of birth of study participants and their parents. Health status was assessed by self-report based on a list of 13 medically treated health problems.^25^ More details about the used scales can be found in eMethods in Supplement 1.

### Statistical Analysis

We conducted mixed-effects multivariable ordered logistic regression analyses adjusted for random effects on study participant level to analyze the association between independent variables and the composite index of self-reported systemic adverse effects (outcome). The statistical model is detailed in eMethods in Supplement 1. The analysis was baseline adjusted and controlled for time of observation. We used the available data set. Coefficients, odds ratios (ORs) and 95% CIs of continuous variables were reported for the difference between 25% and 75% percentile (ie, IQR) in the respective variables (eg, a 3-point difference for expected benefit of vaccination).

Sensitivity analyses identified associations between independent variables and specific outcomes that were reported at any time of observation by at least 10% of the population. These analyses used the same statistical model as the main analysis. Additionally, we provided ORs for unadjusted associations between independent variables and the composite outcome index, which were controlled for baseline values, time of observation, and random effects on the study participant level. The interrelation between the independent variables in the main analysis was analyzed by Pearson correlation. An α level of 5% (2-sided *P* < .05) was defined as statistically significant. The statistical analyses were performed using Stata, version 15.1 (StataCorp LLC).

## Results

The median age of the 1678 study participants was 34 (IQR, 27-44) years (1386 [82.6%] younger than 50 years); 862 (51.4%) were women, 808 (48.2%) were men, and 8 (0.5%) were nonbinary. Most participants (924 [55.1%]) had a tertiary level education; 674 (40.2%) completed secondary level schooling and 79 (4.7%) did not. More than 3 of 4 individuals (1297 [77.3%]) received the BNT162b2 vaccine, compared with 381 (22.7%) receiving the mRNA-1273 vaccine. A complete description of the study population is shown in Table 1.

**Table 1.** Patient Characteristics^a^

Characteristic	Values
Total No. of patients	1678
Age, median (IQR), y	34 (27-44)
Sex	
Women	862 (51.4)
Men	808 (48.2)
Nonbinary	8 (0.5)
Living arrangements	
Living alone	457 (27.2)
Living together with other people	1221 (72.8)
Married or cohabiting	849 (50.6)
Living together with their own children or the children of their partner	451 (26.7)
Living together with their own parents or the parents of their partner	174 (10.4)
Living together with other family members	84 (5.0)
Living together with others (eg, in a shared flat)	141 (8.4)
Educational level (pursuant to CASMIN)	
Higher or lower tertiary education	925 (55.1)
Secondary school certificate or A-level equivalent	675 (40.2)
Inadequately completed, general elementary or basic vocational	78 (4.7)
Migration status	
Study participant and both parents born in Germany	1210 (72.1)
Study participant born in Germany and at least 1 parent born abroad	238 (14.2)
Study participant born abroad	230 (13.7)
Self-reported health problems	
Back pain	382 (22.8)
Depression	228 (13.6)
Gastrointestinal tract symptoms	164 (9.8)
Hypertension	121 (7.2)
Pulmonary disease	91 (5.4)
Rheumatism or other autoimmune disease	79 (4.7)
Osteoarthritis	56 (3.3)
Heart disease	51 (3.0)
Anemia or other blood disease	36 (2.2)
Kidney disease	33 (2.0)
Diabetes	28 (1.7)
Cancer	20 (1.2)
Liver disease	15 (0.9)
Vaccination	
BNT162b2 (Pfizer BioNTech)	1297 (77.3)
mRNA-1273 (Moderna)	381 (22.7)
Anxiety or depression (pursuant to PHQ-4)	
PHQ-4 score, median (IQR)	0 (0-2)
≥3 Points in depression	682 (40.6)
≥3 Points in anxiety	588 (35.0)
Somatosensory Amplification Scale score, median (IQR)	16 (12-20)

Abbreviations: CASMIN, Comparative Analysis of Social Mobility in Industrial Nations; mRNA, messenger RNA; PHQ-4, Patient Health Questionnaire.

^a^ Unless otherwise indicated, data are expressed as No. (%) of participants.

**Figure 1.**
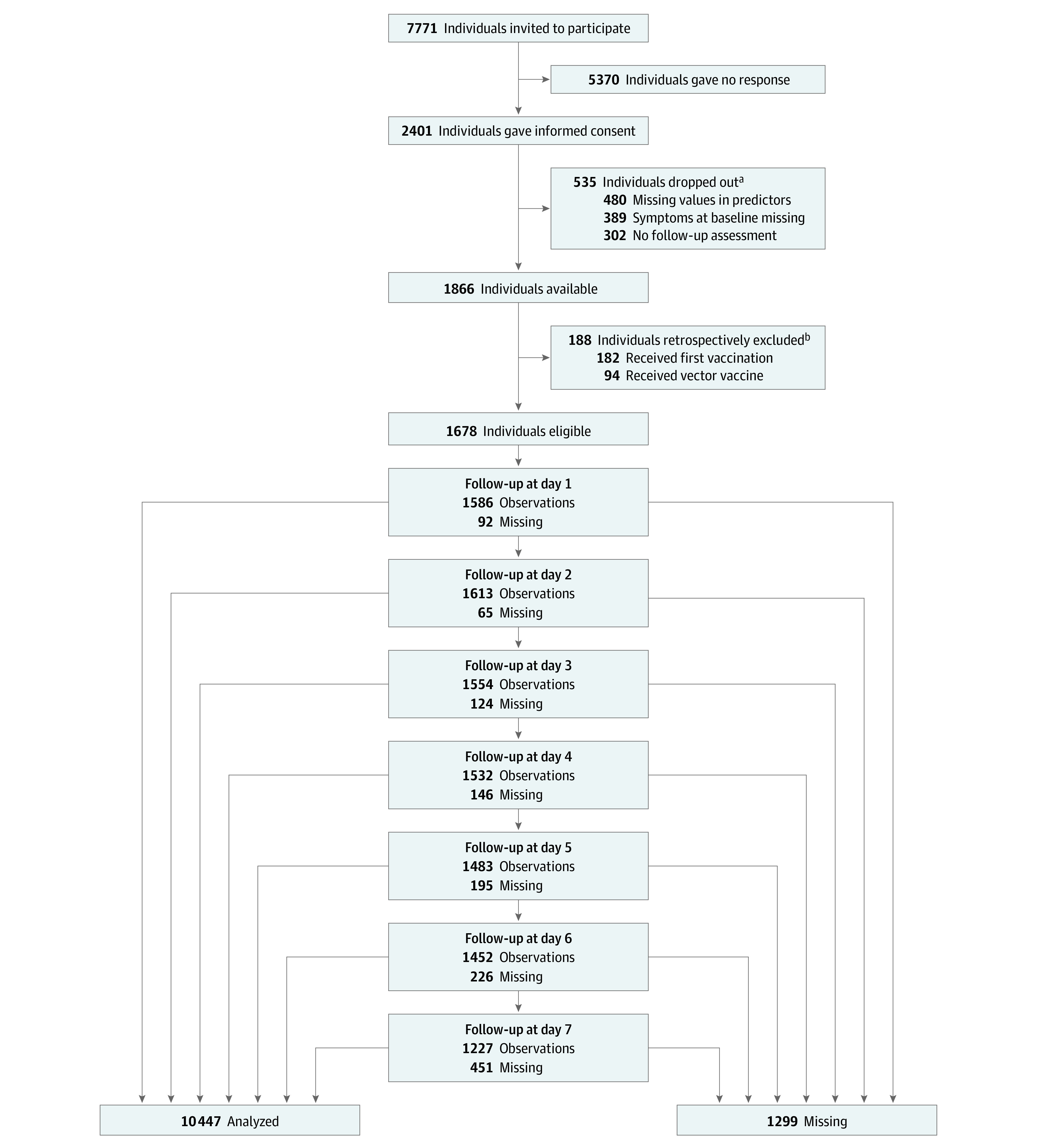
Recruitment of Study Participants and Participation in Follow-up Assessments ^a^The reasons for dropping out are not mutually exclusive. ^b^The reasons for exclusion are not mutually exclusive.

Recruitment and data collection is described in Figure 1. We invited 7771 individuals; 5370 did not respond, 535 provided incomplete information, and 1866 (24.0%) agreed to participate. After exclusion of 188 ineligible participants, 1678 individuals were included in data analysis. Participation in each of the 7 follow-up assessments ranged from 1613 to 1227 individuals. In total, 10 447 observations were analyzed and 1299 (11.1%) were missing. Loss to follow-up comprised 451 individuals (ie, 49 after follow-up 1, 38 after follow-up 2, 33 after follow-up 3, 33 after follow-up 4, 38 after follow-up 5, and 260 after follow-up 6). There were 2 missing values in outcome variables, and no other item nonresponse in the analyzed data. A complete data set was provided by 1096 individuals.

**Figure 2.**
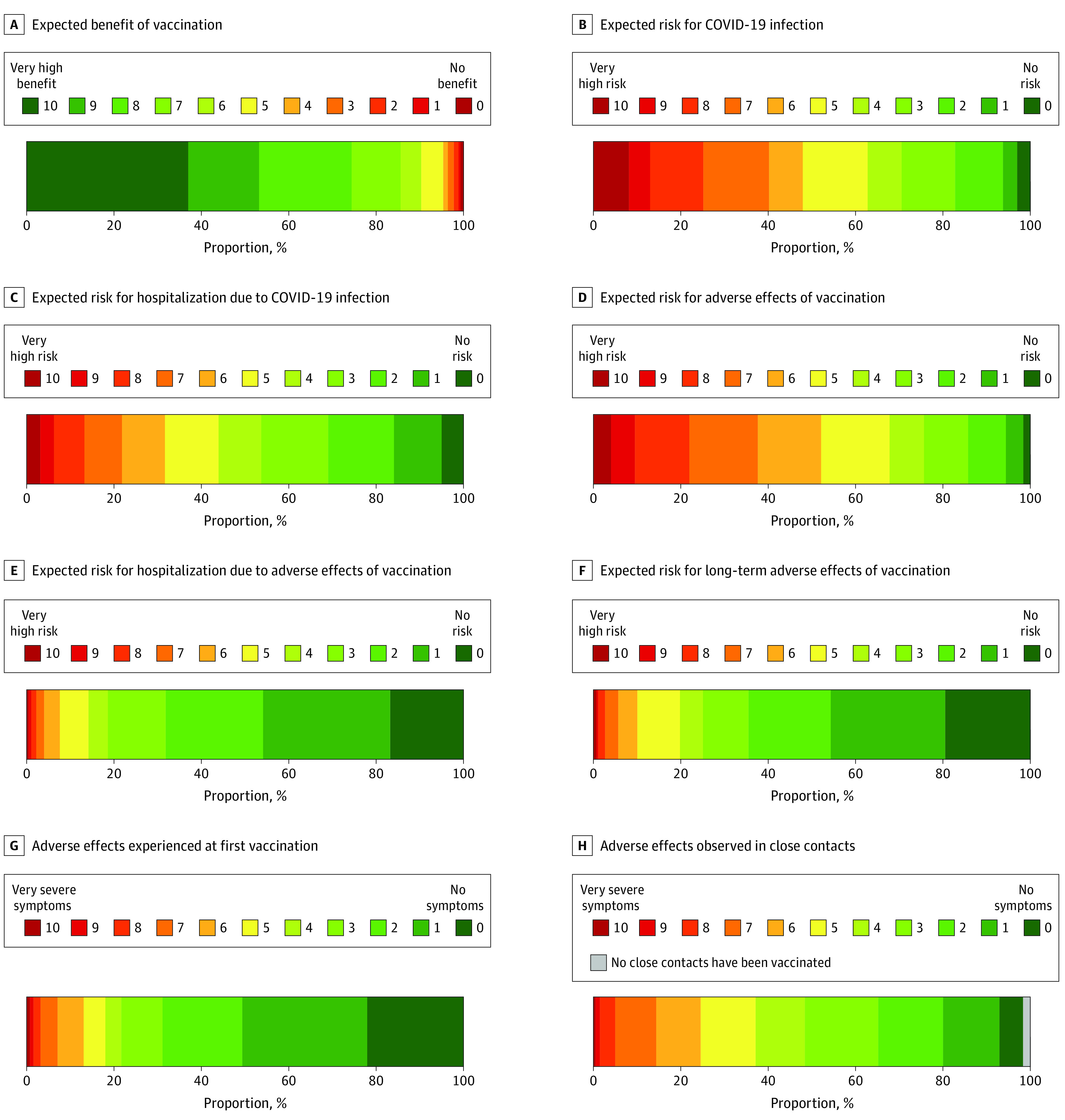
Expected Benefits and Risks of COVID-19 Vaccination (N = 1678)

Perceived benefits and risks related to COVID-19 vaccination are shown in Figure 2. High benefit expectations (6 or more points) were endorsed by 151 (90.3%) of the participants. At the same time, 804 participants (47.9%) expected high risk (6 or more points) for COVID-19 and 531 (31.6%) expected a high risk for COVID-19–related hospitalization. A total of 875 participants (52.1%) expected a high risk for systemic adverse effects, 127 (7.6%) expected a high risk for hospitalization due to adverse effects, and 169 (10.1%) expected a high risk for long-term adverse effects. Severe adverse effects (6 or more points) at the first vaccination had been experienced by 220 participants (13.1%). Severe adverse effects among close contacts had been observed by 412 participants (24.6%), while 28 (1.7%) reported that they did not have close contacts who had already been vaccinated against SARS-CoV-2.

**Figure 3.**
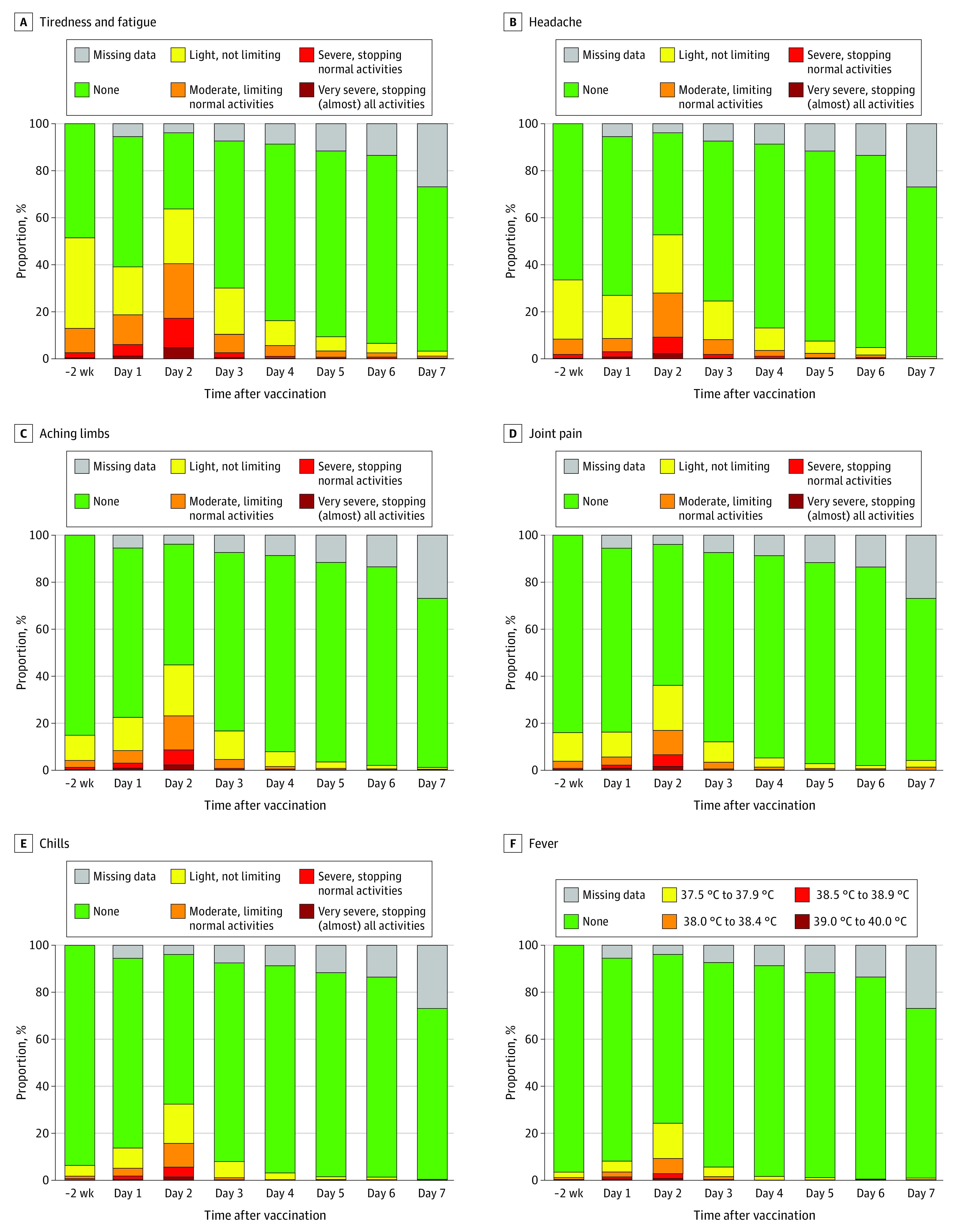
Selected Prevaccine Symptom Levels and Systemic Adverse Effects (N = 1678)

Symptoms in the 2 weeks before vaccination and systemic adverse effects over 7 days are depicted in Figure 3 and eFigure 1 in Supplement 1. Most frequently, study participants reported tiredness or fatigue (1069 [63.7%] with at least light severity on day 2), headache (885 [52.7%]), aching limbs (752 [44.8%]), joint pain (606 [36.1%]), chills (544 [32.4%]) or fever (407 [24.3%]). The distribution of other continuous variables can be found in eFigures 2 to 4 in Supplement 1 and the composite index of systemic adverse effects is shown in eFigure 5 in Supplement 1.

Associations with systemic adverse effects are shown in Table 2. This risk of more severe systemic adverse effects was increased in patients with higher Somatosensory Amplification Scale scores (OR, 1.21 [95% CI, 1.06-1.38]; *P* = .004) and higher levels of anxiety and depression (OR, 1.15 [95% CI, 1.05-1.26]; *P* = .004). Lower expectations about the benefit of vaccination (OR for higher expectations, 0.72 [95% CI, 0.63-0.83]; *P* < .001), higher expectations about adverse effects (OR, 1.39 [95% CI, 1.23-1.58]; *P* < .001), and experience of more severe reactions at first vaccination (OR, 1.60 [95% CI, 1.42-1.82]; *P* < .001) were each associated with higher risk of severe systemic adverse effects. Observed adverse effects in close contacts were not associated with actual risk of systemic adverse effects.

**Table 2.** Associations With Systemic Adverse Effects: Results of Mixed-Effects Ordered Logistic Regression Analysis Adjusted for Random Effects on the Patient Level^a^

Characteristic	OR (95% CI)	*P* value
Highest symptom level in the last 2 weeks before vaccination		
No symptoms in all 12 categories	1 [Reference]	NA
Light symptoms in ≥1 category	1.40 (1.15-1.71)	.001
Moderate symptoms in ≥1 category	1.68 (1.28-2.19)	<.001
Severe symptoms in ≥1 category	2.03 (1.28-3.24)	.003
Very severe symptoms in ≥1 category	3.59 (1.65-7.82)	.001
Time of observation, day after vaccination		
1	1 [Reference]	NA
2	4.98 (4.30-5.78)	<.001
3	0.54 (0.46-0.63)	<.001
4	0.19 (0.16-0.22)	<.001
5	0.09 (0.07-0.11)	<.001
6	0.05 (0.04-0.06)	<.001
7	0.03 (0.02-0.04)	<.001
Age (per 10 y)	0.94 (0.87-1.02)	.14
Sex		
Women	1 [Reference]	NA
Men	0.74 (0.62-0.89)	.001
Nonbinary	1.07 (0.33-3.48)	.91
Living arrangement		
Living together with others	1 [Reference]	NA
Living alone	1.02 (0.84-1.23)	.87
Educational level (pursuant to CASMIN)		
Higher or lower tertiary education	1 [Reference]	NA
Secondary school certificate or A-level equivalent	0.96 (0.80-1.14)	.62
Inadequately completed, general elementary or basic vocational	0.55 (0.35-0.87)	.01
Migration status		
Participant and both parents born in Germany	1 [Reference]	NA
Participant born in Germany and ≥1 parent born abroad	1.03 (0.81-1.32)	.80
Participant born abroad	0.83 (0.64-1.07)	.16
Self-reported health problems		
Back pain	1.34 (1.09-1.66)	.006
Depression	1.22 (0.93-1.61)	.14
Gastrointestinal tract symptoms	1.46 (1.10-1.94)	.01
Hypertension	0.70 (0.49-1.01)	.06
Pulmonary disease	1.72 (1.19-2.49)	.004
Rheumatism or other autoimmune disease	0.85 (0.57-1.27)	.43
Heart disease	1.06 (0.63-1.80)	.82
Osteoarthritis	0.85 (0.52-1.39)	.51
Kidney disease	0.65 (0.34-1.23)	.19
Anemia or other blood disease	1.30 (0.73-2.32)	.38
Diabetes	0.65 (0.33-1.29)	.22
Cancer	1.24 (0.55-2.77)	.61
Liver disease	0.60 (0.23-1.59)	.31
Anxiety or depression (per 2-point difference in PHQ-4 score)	1.15 (1.05-1.26)	.004
Vaccination		
BNT162b2 (Pfizer BioNTech)	1 [Reference]	NA
mRNA-1273 (Moderna)	2.45 (2.01-2.99)	<.001
Somatosensory Amplification Scale score (per 8-point difference)	1.21 (1.06-1.38)	.004
Expected benefit of vaccination (per 3-point difference)	0.72 (0.63-0.83)	<.001
Expected risk for COVID-19 infection (per 3-point difference)	1.04 (0.93-1.16)	.50
Expected risk for hospitalization due to COVID-19 infection (per 3-point difference)	0.91 (0.81-1.04)	.16
Expected risk for adverse effects of vaccination (per 3-point difference)	1.39 (1.23-1.58)	<.001
Expected risk for hospitalization due to adverse effects of vaccination (per 3-point difference)	0.88 (0.73-1.04)	.14
Expected risk for long-term adverse effects of vaccination (per 3-point difference)	0.96 (0.83-1.12)	.64
Adverse effects experienced at first vaccination (per 3-point difference)	1.60 (1.42-1.82)	<.001
Adverse effects observed in close contacts (per 3-point difference)	1.07 (0.95-1.21)	.25

Abbreviations: CASMIN, Comparative Analysis of Social Mobility in Industrial Nations; OR, odds ratio; NA, not applicable; PHQ-4, Patient Health Questionnaire.

^a^ Includes 1678 participants and 10 447 observations.

A correlation matrix of independent variables is shown in eFigure 6 in Supplement 1. Nine of the 840 analyzed pairs of variables had a correlation coefficient of greater than 0.30. The highest correlation was found between expected risk for hospitalization due to vaccination and expected long-term adverse effects (*r* = 0.61).

Unadjusted associations between independent variables and the composite outcome can be found in eTables 1 to 10 in Supplement 1. In these analyses, systemic adverse effects were associated with expected benefit of vaccination (OR, 0.74 [95% CI, 0.65-0.86]; *P* < .001), expected risk for hospitalization due to COVID-19 infection (OR, 0.88 [95% CI, 0.79-0.99]; *P* = .03), expected risk for adverse effects (OR, 1.86 [95% CI, 1.66-2.10]; *P* < .001), long-term adverse effects of vaccination (OR, 1.16 [95% CI, 1.02-1.32]; *P* = .03), adverse effects experienced at first vaccination (OR, 2.07 [95% CI, 1.84-2.34]; *P* < .001), adverse effects observed in close contacts (OR, 1.45 [95% CI, 1.28-1.64]; *P* < .001), anxiety and depression levels (OR, 1.33 [95% CI, 1.21-1.46]; *P* < .001), and Somatosensory Amplification Scale score (OR, 1.75 [95% CI, 1.53-1.99]; *P* < .001). Expected risks for COVID-19 infection and for hospitalization due to adverse effects were not associated with symptom burden.

Adjusted models identifying associations between independent variables and 7 specific adverse effects are shown in eTables 11 to 17 in Supplement 1. Consistently, lower expected benefit of vaccination (ORs for higher expected benefit, 0.71 [95% CI, 0.57-0.88; *P* = .002] to 0.80 [95% CI, 0.68-0.95; *P* = .008]) and more severe adverse effects experienced at first vaccination (ORs, 1.25 [95% CI, 1.06-1.47; *P* = .008] to 1.66 [95% CI, 1.38-1.99; *P* < .001]) were associated with higher symptom burden. Additionally, 5 symptoms were associated with higher expected risk for adverse effects (ORs, 1.24 [95% CI, 1.04-1.47; *P* = .01] to 1.39 [95% CI, 1.22-1.58]; *P* < .001) and higher level of anxiety and depression (ORs, 1.12 [95% CI, 1.01-1.24; *P* = .04] to 1.20 [95% CI, 1.02-1.41; *P* = .03]); 2 symptoms were associated with a higher Somatosensory Amplification Scale score (ORs, 1.20 [95% CI, 1.04-1.37; *P* = .01] and 1.26 [95% CI, 1.09-1.47; *P* = .002]); and 1 symptom was associated with lower expected risk for hospitalization due to COVID-19 infection (OR, 0.87 [95% CI, 0.77-0.99]; *P* = .04), hospitalization due to vaccination (OR, 0.81 [95% CI, 0.67-0.97]; *P* = .02), and more severe adverse effects observed in close contacts (OR, 1.15 [95% CI, 1.01-1.30]; *P* = .03). Expected risks for COVID-19 infection and for long-term adverse effects were not associated with specific adverse effects.

## Discussion

In agreement with a priori specified hypotheses, participants who expected a lower benefit from vaccination, those who expected more adverse effects of vaccine, and those who had experienced more adverse effects after first vaccination reported higher severity of most systemic adverse effects after adjusting for prevaccine symptom levels. Likewise, individuals reporting higher scores in anxiety and depression and those scoring higher on the Somatosensory Amplification Scale reported more symptoms in some observed categories. Generally, more severe symptoms were reported by women. Contrary to our hypothesis, observing adverse effects of vaccine in others was not associated with personal systemic adverse effects.

Nocebo effects after vaccination can be caused by 3 main mechanisms: learning, expectations, and misattribution.^26^ Our results suggest the presence of all these mechanisms in our sample.

Symptoms after the first vaccination had the strongest association with adverse effects in our model, indicating the presence of learning mechanisms from prior experiences as a conditioning phenomenon. Such learning mechanisms were already proposed in a comparison of vaccine and placebo groups of the phase 3 trials of mRNA vaccines, where following the second dose, an increase of all adverse effects except local pain could be observed.^27^ However, it is also possible that a certain immunological predisposition regarding adverse effects exists in some patients, which could be responsible for experiencing similar adverse effects in both vaccinations.

Additionally, perceived individual risk for adverse effects indicated negative expectations as another trigger for nocebo effects. Another study that investigated nocebo effects in vaccination-naive participants^28^ also identified that negative expectations, as well as worries about COVID-19 and depressive symptoms, as associated with adverse effects. In our sample, worries about COVID-19 were not a significant factor.

We also observed that the risk for adverse effects increased with the severity of baseline symptoms, indicating misattribution of preexisting symptoms. Our results are in accordance with other studies reporting higher levels of anxiety, depression, and somatosensory amplification as predisposing factors of higher somatic symptom load.^20^ The involved mechanisms include cognitive, emotional, and behavioral factors such as selective attention toward interoceptive cues, amplified perception of benign bodily sensations, and maintenance of these processes through catastrophizing cognitive interpretations and unhelpful illness behaviors. In addition, we observed that a belief in the personal benefit of vaccination was inversely associated with systemic vaccine adverse effects, whereas neither the perceived risk to contract COVID-19 nor the perceived risk for hospitalization due to COVID-19 were associated with vaccine adverse effects.

### Implications for Clinical Practice

In contrast to other predisposing factors for adverse effects like preexisting symptoms, prior experiences, or sex that are documented in our data as well as in other studies,^28^ expectations might be changed via short and economic psychological interventions.^29,30^ Probable effective strategies include framing of adverse effects information by emphasizing the probability of being free from adverse effects,^31^ by elaborating on anticipated positive effects and their working mechanisms,^32^ or by accompanying adverse effect information with specific coping strategies.^29^

Furthermore, counteracting symptom misattribution by explicitly informing about nocebo effects (ie, “Worrying about potential adverse effects can intensify concerns like a self-fulfilling prophecy. People taking placebos in clinical trials often report adverse effects, thereby, misattributing benign bodily sensations such as common headaches or fatigue to the vaccine”) has been shown to be effective in other contexts in reducing experimentally induced nocebo effects^33^ and in functionally adapting patients’ informational needs regarding medication.^34^ Furthermore, patients’ prior experiences and psychosocial characteristics such as the tendency to amplify bodily sensations might be included in contextualized informed consent procedures.^35^

### Limitations and Strengths

This study has some limitations. Our results identify approaches for improving patient information by avoiding triggering potential nocebo effects. However, to evaluate their effectiveness, these approaches must be tested in randomized clinical studies. Furthermore, we did not conduct a sample size calculation; therefore associations with systemic adverse effects could have been missed.

Many COVID-19 studies and mobile application–based studies have large sample sizes (n > 1000), but seemingly low response rates (eg, 12% in Ayoubkhani et al^36^ and 2.4% in Ryan et al^37^). In our case, the response rate of 24.0% is related to high throughput of the vaccination center, in which communication with individuals cannot be as detailed as in other studies. Therefore, response rates are lowered by individuals who were invited to participate but were not eligible (eg, no smartphone, vector vaccines, or first vaccination). Nevertheless, as in other studies, this can limit representativeness if the sample systematically deviates from the population in variables that are associated with the dependent variable, but not included as covariate in the statistical models. For example, one might overestimate nocebo effects if individuals with lower tendency to somatize risk expectations had been less likely to participate.

The low number of participants without secondary education and the high number of participants with tertiary education indicate that our sample is not fully representative of the general population. This can at least in part be explained by the frequently reported uneven distribution of willingness to get vaccinated and time of vaccination across educational levels.^38,39,40^ The distribution in this variable might also correspond with education-based differences in general willingness to participate in scientific studies.^41,42^ Furthermore, 82.6% were younger than 50 years and expectations of symptom load after COVID-19 vaccination might differ in older groups. Also, we did not adjust for possible clusters of vaccinated individuals (eg, family or social ties).

Moreover, we used a composite index as end point that ignores differences between symptoms and neglects multiplicative burden of multiple symptoms. Possible bias resulting from this decision was explored by sensitivity analyses using frequent adverse effects as alternative end points. Importantly, these additional analyses confirmed our main results for most symptoms.

A particular strength of our study is high-data quality. Daily assessments allowed participants to report symptoms within their respective environments with repeated measures capturing natural fluctuations. Analyses were adjusted for prevaccine symptoms, but despite short recall periods,^43,44^ recall bias could not be ruled out completely. Missing observations were not imputed, but our statistical methods facilitated including patients with incomplete assessments. We were also able to analyze adverse effects over all follow-ups and to adjust for possible confounding.

## Conclusions

In this prospective cohort study, severity of systemic adverse effects in the first week after COVID-19 vaccination was not only caused by vaccine-specific reactogenicity but also by psychosocial context factors that can be identified prior to vaccination. We identified 3 major contributors to nocebo effects, namely, personal prior experiences from the first COVID-19 vaccination, individual expectations regarding potential benefits and harms of vaccination, and symptom misattribution. Clinician-patient interactions and public vaccine campaigns may both benefit from these insights by optimizing and contextualizing information provided about COVID-19 vaccines. Unfavorable nocebo-related adverse effects could then be prevented, and overall vaccine acceptance could be improved.

## Notes

Supplement 1. **eMethods.** Outcome and Independent Variables and Statistical Analysis
eReferences
**eFigure 1.** Selected Prevaccine Symptom Levels and Systemic Adverse Effects (N = 1678) **eFigure 2.** Histogram and Kernel Density Estimation of Age (N = 1678) **eFigure 3.** Histogram of PHQ-4 Score (N = 1678) **eFigure 4.** Histogram and Kernel Density Estimation of Somatosensory Amplification Scale Score (N = 1678) **eFigure 5.** Composite Severity Score of Baseline Symptom Load and Systemic Adverse Effects in Any of the 12 Categories (N = 1678) **eFigure 6.** Correlation Matrix of Independent Variables in Main Analysis (N = 10 447) **eTable 1.** Unadjusted Association Between Expected Benefit of Vaccination and Systemic Adverse Effects: Results of Mixed-Effects Ordered Logistic Regression Analysis Adjusted for Random Effects on the Patient Level **eTable 2.** Unadjusted Association Between Expected Risk for COVID-19 Infection and Systemic Adverse Effects: Results of Mixed-Effects Ordered Logistic Regression Analysis Adjusted for Random Effects on the Patient Level **eTable 3.** Unadjusted Association Between Expected Risk for Hospitalization due to COVID-19 Infection and Systemic Adverse Effects: Results of Mixed-Effects Ordered Logistic Regression Analysis Adjusted for Random Effects on the Patient Level **eTable 4.** Unadjusted Association Between Expected Risk for Adverse Effects of Vaccination and Systemic Adverse Effects: Results of Mixed-Effects Ordered Logistic Regression Analysis Adjusted for Random Effects on the Patient Level **eTable 5.** Unadjusted Association Between Expected Risk for Hospitalization Due to Adverse Effects of Vaccination and Systemic Adverse Effects: Results of Mixed-Effects Ordered Logistic Regression Analysis Adjusted for Random Effects on the Patient Level **eTable 6.** Unadjusted Association Between Expected Risk for Long-term Adverse Effects of Vaccination and Systemic Adverse Effects: Results of Mixed-Effects Ordered Logistic Regression Analysis Adjusted for Random Effects on the Patient Level **eTable 7.** Unadjusted Association Between Adverse Effects Experienced at First Vaccination and Systemic Adverse Effects: Results of Mixed-Effects Ordered Logistic Regression Analysis Adjusted for Random Effects on the Patient Level **eTable 8.** Unadjusted Association Between Adverse Effects Observed in Close Contacts and Systemic Adverse Effects: Results of Mixed-Effects Ordered Logistic Regression Analysis Adjusted for Random Effects on the Patient Level **eTable 9.** Unadjusted Association Between Anxiety and Depression and Systemic Adverse Effects: Results of Mixed-Effects Ordered Logistic Regression Analysis Adjusted for Random Effects on the Patient Level **eTable 10.** Unadjusted Association Between Somatosensory Amplification and Systemic Adverse Effects: Results of Mixed-Effects Ordered Logistic Regression Analysis Adjusted for Random Effects on the Patient Level **eTable 11.** Associations With Tiredness/Fatigue: Results of Mixed-Effects Ordered Logistic Regression Analysis Adjusted for Random Effects on the Patient Level **eTable 12.** Associations With Headache: Results of Mixed-Effects Ordered Logistic Regression Analysis Adjusted for Random Effects on the Patient Level **eTable 13.** Associations With Aching Limbs: Results of Mixed-Effects Ordered Logistic Regression Analysis Adjusted for Random Effects on the Patient Level **eTable 14.** Associations With Joint Pain: Results of Mixed-Effects Ordered Logistic Regression Analysis Adjusted for Random Effects on the Patient Level **eTable 15.** Associations With Chills: Results of Mixed-Effects Ordered Logistic Regression Analysis Adjusted for Random Effects on the Patient Level **eTable 16.** Associations With Fever: Results of Mixed-Effects Ordered Logistic Regression Analysis Adjusted for Random Effects on the Patient Level **eTable 17.** Associations With Deep Leg Pain: Results of Mixed-Effects Ordered Logistic Regression Analysis Adjusted for Random Effects on the Patient Level **eBox 1.** Symptom Diary—English Version, Translated From German **eBox 2.** Symptom Diary—German Version

Supplement 2.
Data Sharing Statement
