# Supplementary material for: Expectations and Prior Experiences Associated With Adverse Effects of COVID-19 Vaccination
Source: JAMA Netw Open. 2023 Mar 27;6(3):e234732. doi: 10.1001/jamanetworkopen.2023.4732 (PMC10043751; doi:10.1001/jamanetworkopen.2023.4732)
Supplement: Supplement 1. — eMethods. Outcome and Independent Variables and Statistical Analysis eReferences eFigure 1. Selected Prevaccine Symptom Levels and Systemic Adverse Effects (N = 1678) eFigure 2. Histogram and Kernel Density Estimation of Age (N = 1678) eFigure 3. Histogram of PHQ-4 Score (N = 1678) eFigure 4. Histogram and Kernel Density Estimation of Somatosensory Amplification Scale Score (N = 1678) eFigure 5. Composite Severity Score of Baseline Symptom Load and Systemic Adverse Effects in Any of the 12 Categories (N = 1678) eFigure 6. Correlation Matrix of Independent Variables in Main Analysis (N = 10 447) eTable 1. Unadjusted Association Between Expected Benefit of Vaccination and Systemic Adverse Effects: Results of Mixed-Effects Ordered Logistic Regression Analysis Adjusted for Random Effects on the Patient Level eTable 2. Unadjusted Association Between Expected Risk for COVID-19 Infection and Systemic Adverse Effects: Results of Mixed-Effects Ordered Logistic Regression Analysis Adjusted for Random Effects on the Patient Level eTable 3. Unadjusted Association Between Expected Risk for Hospitalization due to COVID-19 Infection and Systemic Adverse Effects: Results of Mixed-Effects Ordered Logistic Regression Analysis Adjusted for Random Effects on the Patient Level eTable 4. Unadjusted Association Between Expected Risk for Adverse Effects of Vaccination and Systemic Adverse Effects: Results of Mixed-Effects Ordered Logistic Regression Analysis Adjusted for Random Effects on the Patient Level eTable 5. Unadjusted Association Between Expected Risk for Hospitalization Due to Adverse Effects of Vaccination and Systemic Adverse Effects: Results of Mixed-Effects Ordered Logistic Regression Analysis Adjusted for Random Effects on the Patient Level eTable 6. Unadjusted Association Between Expected Risk for Long-term Adverse Effects of Vaccination and Systemic Adverse Effects: Results of Mixed-Effects Ordered Logistic Regression Analysis Adjusted for Random Effects on the Patient Lev [file jamanetwopen-e234732-s001.pdf]

Supplementary Online Content

Schäfer I, Oltrogge JH, Nestoriuc Y, et al. Expectations and prior experiences associated with adverse effects of COVID-19 vaccination. *JAMA Netw Open*. 2023;6(3):e234732. doi:10.1001/jamanetworkopen.2023.4732

eMethods. Outcome and Independent Variables and Statistical Analysis
eReferences
eFigure 1. Selected Prevaccine Symptom Levels and Systemic Adverse Effects (N = 1678)
eFigure 2. Histogram and Kernel Density Estimation of Age (N = 1678)
eFigure 3. Histogram of PHQ-4 Score (N = 1678)
eFigure 4. Histogram and Kernel Density Estimation of Somatosensory Amplification Scale Score (N = 1678)
eFigure 5. Composite Severity Score of Baseline Symptom Load and Systemic Adverse Effects in Any of the 12 Categories (N = 1678)
eFigure 6. Correlation Matrix of Independent Variables in Main Analysis (N = 10 447)
eTable 1. Unadjusted Association Between Expected Benefit of Vaccination and Systemic Adverse Effects: Results of Mixed-Effects Ordered Logistic Regression Analysis Adjusted for Random Effects on the Patient Level
eTable 2. Unadjusted Association Between Expected Risk for COVID-19 Infection and Systemic Adverse Effects: Results of Mixed-Effects Ordered Logistic Regression Analysis Adjusted for Random Effects on the Patient Level
eTable 3. Unadjusted Association Between Expected Risk for Hospitalization due to COVID-19 Infection and Systemic Adverse Effects: Results of Mixed-Effects Ordered Logistic Regression Analysis Adjusted for Random Effects on the Patient Level
eTable 4. Unadjusted Association Between Expected Risk for Adverse Effects of Vaccination and Systemic Adverse Effects: Results of Mixed-Effects Ordered Logistic Regression Analysis Adjusted for Random Effects on the Patient Level
eTable 5. Unadjusted Association Between Expected Risk for Hospitalization Due to Adverse Effects of Vaccination and Systemic Adverse Effects: Results of Mixed-Effects Ordered Logistic Regression Analysis Adjusted for Random Effects on the Patient Level
eTable 6. Unadjusted Association Between Expected Risk for Long-term Adverse Effects of Vaccination and Systemic Adverse Effects: Results of Mixed-Effects Ordered Logistic Regression Analysis Adjusted for Random Effects on the Patient Level
eTable 7. Unadjusted Association Between Adverse Effects Experienced at First Vaccination and Systemic Adverse Effects: Results of Mixed-Effects Ordered Logistic Regression Analysis Adjusted for Random Effects on the Patient Level
eTable 8. Unadjusted Association Between Adverse Effects Observed in Close Contacts and Systemic Adverse Effects: Results of Mixed-Effects Ordered Logistic Regression Analysis Adjusted for Random Effects on the Patient Level
eTable 9. Unadjusted Association Between Anxiety and Depression and Systemic Adverse Effects: Results of Mixed-Effects Ordered Logistic Regression Analysis Adjusted for Random Effects on the Patient Level
eTable 10. Unadjusted Association Between Somatosensory Amplification and Systemic Adverse Effects: Results of Mixed-Effects Ordered Logistic Regression Analysis Adjusted for Random Effects on the Patient Level
eTable 11. Associations With Tiredness/Fatigue: Results of Mixed-Effects Ordered Logistic Regression Analysis Adjusted for Random Effects on the Patient Level
eTable 12. Associations With Headache: Results of Mixed-Effects Ordered Logistic Regression Analysis Adjusted for Random Effects on the Patient Level

© 2023 Schäfer I et al. *JAMA Network Open*.

eTable 13. Associations With Aching Limbs: Results of Mixed-Effects Ordered Logistic Regression Analysis Adjusted for Random Effects on the Patient Level
eTable 14. Associations With Joint Pain: Results of Mixed-Effects Ordered Logistic Regression Analysis Adjusted for Random Effects on the Patient Level
eTable 15. Associations With Chills: Results of Mixed-Effects Ordered Logistic Regression Analysis Adjusted for Random Effects on the Patient Level
eTable 16. Associations With Fever: Results of Mixed-Effects Ordered Logistic Regression Analysis Adjusted for Random Effects on the Patient Level
eTable 17. Associations With Deep Leg Pain: Results of Mixed-Effects Ordered Logistic Regression Analysis Adjusted for Random Effects on the Patient Level
eBox 1. Symptom Diary—English Version, Translated From German
eBox 2. Symptom Diary—German Version

This supplementary material has been provided by the authors to give readers additional information about their work.

© 2023 Schäfer I et al. *JAMA Network Open*.

eMethods. Outcome and Independent Variables and Statistical Analysis

Outcome variables

Daily ratings included twelve symptom areas with a severity rating on a five to six-item Likert scale: (1) Aching limbs, (2) chills, (3) deep leg pain, (4) headache, (5) heart pain, (6) joint pain, (7) shortness of breath and (8) tiredness/fatigue were rated on the levels (a) none, (b) light/not limiting, (c) moderate/limiting normal activities, (d) severe/stopping normal activities and (e) very severe/stopping (almost) all activities. (9) Fever was reported on the levels (a) none, (b) 37.5°C to 37.9°C, (c) 38.0°C to 38.4°C, (d) 38.5°C to 38.9°C, (e) 39.0°C to 40.0°C, and (f) more than 40.0°C. The rating of (10) diarrhea was based on the levels (a) none or less than two times soft stool in 24 hours, (b) two or three times in 24 hours, (c) four or five times in 24 hours, (d) six or more times in 24 hours, and (e) six or more times in 24 hours, medical clarification required. The rating of (11) vomiting included the levels (a) none, (b) one or two times vomiting in 24 hours, (c) more than twice in 24 hours, (d) fluid substitution required, (e) fluid substitution and medical clarification required. (12) Hematomas/punctiform hemorrhages were rated on the levels (a) none, (b) one to five hematomas or punctiform hemorrhages, (c) six to ten, (d) more than ten, and (e) more than ten, medical clarification required. In order to use only variables with five levels for the calculation of the composite endpoint, we combined the two highest categories in the symptom area "fever".

Independent variables

The study participants used a numerical rating scale ranging from 0 = "no benefit" to 10 = "very high benefit" to rate (1) the expected benefit of the vaccination. A scale ranging from 0 = "no risk" to 10 = "very high risk" was used to report the expected risks for (2) COVID-19 infection (without vaccination), (3) hospitalization due to COVID-19 infection (without vaccination), (4) systemic side effects, (5) hospitalization due to side effects, and (6) long-term side effects of vaccination. Additionally, (7) side effects experienced at the first vaccination and (8) side effects observed in close contacts were reported on a scale from 0 = "no complaints" to 10 = "very severe complaints". Participants, who did not have close contacts who received a vaccination were treated as people who observed no complaints in close contacts.

The Patient Health Questionnaire PHQ-4 consists of two items each measuring the frequency of anxiety and depression symptoms on a four-point Likert scale from "not at all" to "almost every day". Good validity and reliability have been demonstrated for both scales. On both scales, three points are standard cut-off values, respectively [1]. The Somatosensory Amplification Scale consists of ten items, each rated on a five- point Likert scale according to whether it is "characteristic of you in general" from "not at all" to "extremely". Validity and reliability of the Somatosensory Amplification Scale have been confirmed [2-3]. For our multivariable analyses, living arrangements were dichotomized into "living alone" and "living together with other people".

Statistical methods

The distribution of continuous variables in our model is reported by histograms and kernel density estimators. We conducted mixed-effects multivariable ordered logistic regression analyses adjusted for random effects on the study participant level to analyze the association between expected risks and benefits of the vaccination, observed and experienced systemic side effects as well as "anxiety", "depression", and "somatosensory amplification" (continuous independent variables) and the composite index of self-reported systemic side effects (ordinally scaled outcome).

© 2023 Schäfer I et al. *JAMA Network Open*.

The statistical model included the potential confounders

- "age" (interval scaled; coefficient and confidence interval reported for 10 points difference),
- "sex" (nominally scaled and analyzed with "female" as reference category and "male" and "non-binary" as dummy variables),
- the dichotomized "living arrangement" (nominally scaled),
- "educational level" (nominally scaled and analyzed with "tertiary education" as reference and "inadequately completed, general elementary or basic vocational" and "secondary school certificate or A level equivalent" as dummy variables),
- "migrant status" (nominally scaled and analyzed with "participant and both parents born in Germany" as reference and "participant born in Germany and at least one parent born abroad" and "participant born abroad" as dummy variables"),
- "self-reported health problems" (nominally scaled dichotomous variables), and
- "vaccine given at the day of recruitment" (nominally scaled dichotomous variable).

The analysis was baseline-adjusted for pre-vaccine symptom levels (nominally scaled and analyzed with "no symptoms in all twelve categories" as reference and four dummy variables representing "light", "moderate", "severe", and "very severe symptoms at least one category", respectively) and controlled for the time of observation (nominally scaled and analyzed with "day one" as reference category and six dummy variables representing "day two" to "day seven", respectively).

eReferences

1. Löwe B, Wahl I, Rose M, et al. A 4-item measure of depression and anxiety: Validation and standardization of the Patient Health Questionnaire-4 (PHQ-4) in the general population. J Affect Disord 2010;122:86–95. doi:10.1016/j.jad.2009.06.019

2. Doering BK, Nestoriuc Y, Barsky AJ, et al. Is somatosensory amplification a risk factor for an increased report of side effects? Reference data from the German general population. J Psychosom Res 2015;79:492–7. doi: 10.1016/j.jpsychores.2015.10.010.

3. Speckens AE, Spinhoven P, Sloekers PP, et al. A validation study of the Whitely Index, the Illness Attitude Scales, and the Somatosensory Amplification Scale in general medical and general practice patients. J Psychosom Res 1996;40:95–104. doi: 10.1016/0022-3999(95)00561-7.

© 2023 Schäfer I et al. *JAMA Network Open*.

eFigure 1. Selected Prevaccine Symptom Levels and Systemic Adverse Effects (N = 1678)

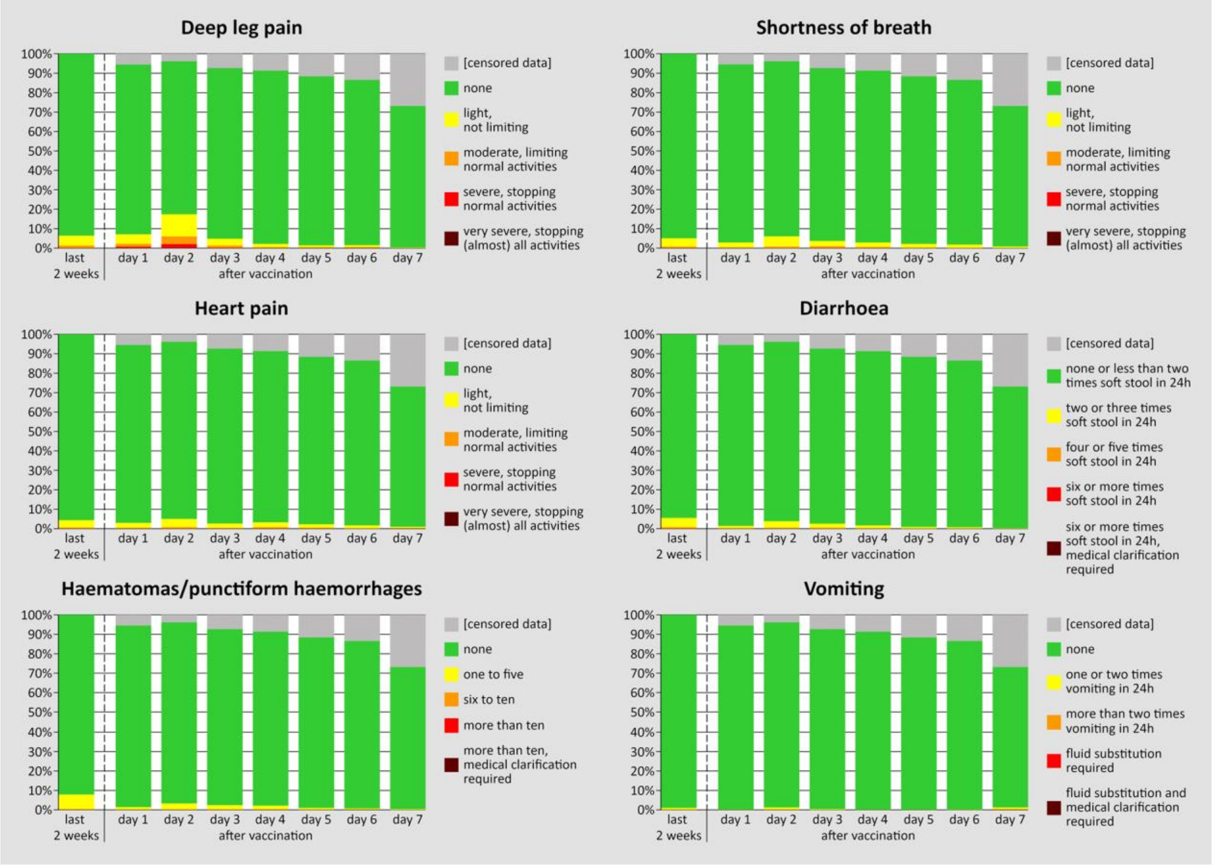

eFigure 2. Histogram and Kernel Density Estimation of Age (N = 1678)

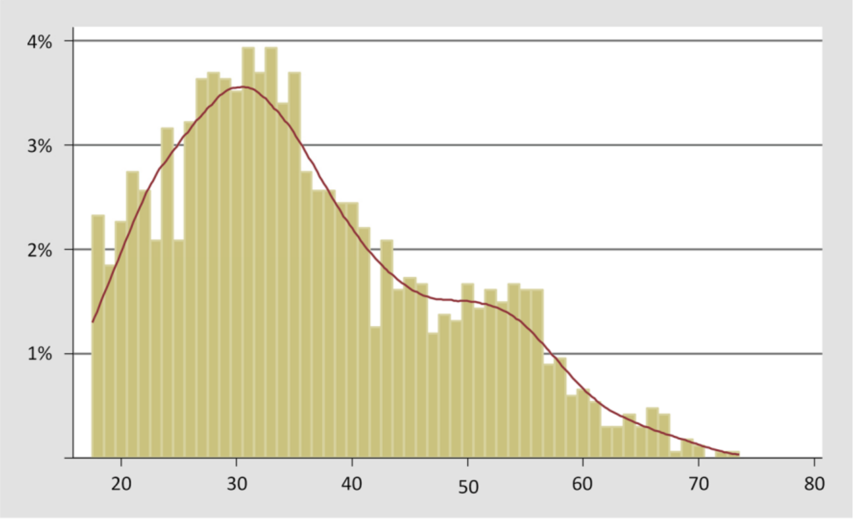

© 2023 Schäfer I et al. *JAMA Network Open*.

eFigure 3. Histogram of PHQ-4 Score (N = 1678)

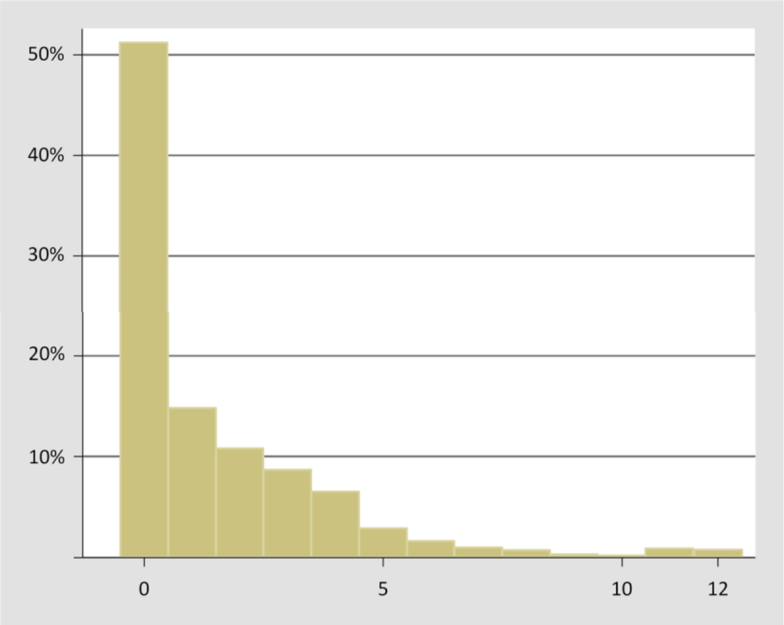

eFigure 4. Histogram and Kernel Density Estimation of Somatosensory Amplification Scale Score (N = 1678)

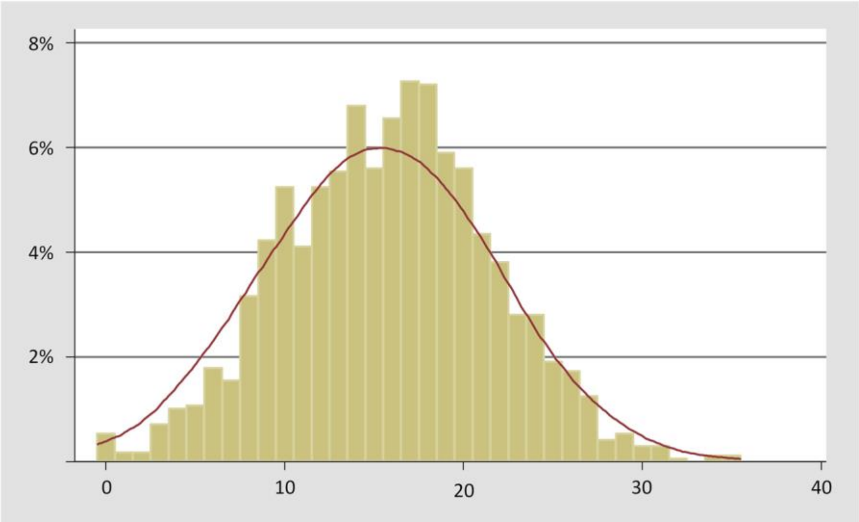

eFigure 5. Composite Severity Score of Baseline Symptom Load and Systemic Adverse Effects in Any of the 12 Categories (N = 1678)

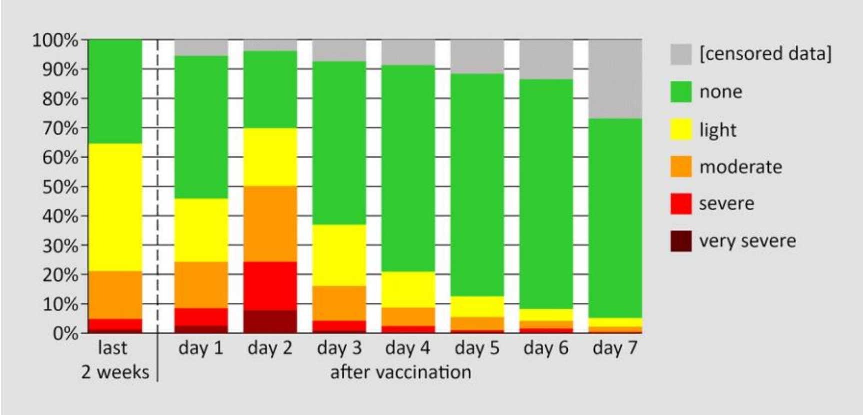

© 2023 Schäfer I et al. *JAMA Network Open*.

eFigure 6. Correlation Matrix of Independent Variables in Main Analysis (N = 10 447)

	Light symptoms at baseline	Moderate symptoms at baseline	Severe symptoms at baseline	Very severe symptoms at baseline	Day two after vaccination	Day three after vaccination	Day four after vaccination	Day five after vaccination	Day six after vaccination	Day seven after vaccination	Age	Sex: female	Sex: non-binary	Living arrangement: living alone	Education: secondary	Education: tertiary	Migration: parent born abroad	Migration: participant born abroad	Health problem: back pain	Health problem: depression	Health problem: gastrointestinal
Light symptoms at baseline	1.00																				
Moderate symptoms at baseline	-0.39	1.00																			
Severe symptoms at baseline	-0.17	-0.09	1.00																		
Very severe symptoms at baseline	-0.10	-0.05	-0.02	1.00																	
Day two after vaccination	0.00	0.00	0.00	0.00	1.00																
Day three after vaccination	0.00	0.00	0.00	0.00	-0.17	1.00															
Day four after vaccination	0.00	0.00	0.00	0.00	-0.17	-0.17	1.00														
Day five after vaccination	0.00	0.00	0.00	0.00	-0.17	-0.17	-0.17	1.00													
Day six after vaccination	0.00	0.00	0.00	0.00	-0.17	-0.17	-0.17	-0.17	1.00												
Day seven after vaccination	0.00	0.00	0.00	0.00	-0.17	-0.17	-0.17	-0.17	-0.17	1.00											
Age	-0.06	-0.08	-0.05	-0.02	0.00	0.00	0.00	0.00	0.00	0.00	1.00										
Sex: female	-0.06	-0.04	-0.05	-0.03	0.00	0.00	0.00	0.00	0.00	0.00	0.07	1.00									
Sex: non-binary	-0.01	0.04	-0.01	-0.01	0.00	0.00	0.00	0.00	0.00	0.00	-0.04	-0.07	1.00								
Living arrangement: living alone	-0.05	-0.01	-0.01	0.02	0.00	0.00	0.00	0.00	0.00	0.00	0.10	0.09	0.00	1.00							
Education: secondary	0.01	0.00	0.00	0.03	0.00	0.00	0.00	0.00	0.00	0.00	0.00	0.02	0.08	0.07	1.00						
Education: tertiary	0.01	0.00	0.00	-0.04	0.00	0.00	0.00	0.00	0.00	0.00	-0.02	-0.04	-0.08	-0.08	-0.91	1.00					
Migration: parent born abroad	0.03	0.02	0.02	-0.03	0.00	0.00	0.00	0.00	0.00	0.00	-0.14	-0.04	0.02	-0.03	0.03	-0.01	1.00				
Migration: participant born abroad	0.00	-0.01	0.02	0.07	0.00	0.00	0.00	0.00	0.00	0.00	-0.06	-0.01	0.00	-0.01	-0.09	0.06	-0.16	1.00			
Health problem: back pain	0.03	0.07	0.03	0.03	0.00	0.00	0.00	0.00	0.00	0.00	0.05	-0.05	0.00	0.01	0.09	-0.12	-0.03	0.02	1.00		
Health problem: depression	0.03	0.11	0.03	0.02	0.00	0.00	0.00	0.00	0.00	0.00	-0.09	-0.11	0.12	0.10	0.13	-0.13	0.00	-0.01	0.18	1.00	
Health problem: gastrointestinal	0.03	0.08	0.04	-0.02	0.00	0.00	0.00	0.00	0.00	0.00	0.00	-0.11	-0.02	0.02	0.02	-0.03	0.03	-0.04	0.24	0.12	1.00
Health problem: hypertension	-0.03	0.03	0.01	0.05	0.00	0.00	0.00	0.00	0.00	0.00	0.18	0.05	-0.02	0.04	0.07	-0.09	-0.05	-0.02	0.10	0.04	0.08
Health problem: pulmonary	-0.03	0.06	0.07	0.02	0.00	0.00	0.00	0.00	0.00	0.00	0.02	-0.01	-0.02	0.00	0.03	-0.05	0.03	-0.03	0.13	0.08	0.11
Health problem: rheumatism	-0.02	0.02	0.00	0.03	0.00	0.00	0.00	0.00	0.00	0.00	0.13	-0.08	0.03	0.03	0.02	-0.02	-0.02	0.01	0.01	0.05	0.05
Health problem: heart	-0.04	0.06	-0.02	0.01	0.00	0.00	0.00	0.00	0.00	0.00	0.14	0.07	-0.01	0.06	0.06	-0.08	-0.01	-0.02	0.10	0.03	0.09
Health problem: osteoarthritis	-0.04	0.08	0.02	0.04	0.00	0.00	0.00	0.00	0.00	0.00	0.17	-0.03	-0.01	0.01	0.04	-0.07	-0.04	-0.01	0.09	0.06	0.05
Health problem: renal disease	-0.03	0.04	0.02	0.06	0.00	0.00	0.00	0.00	0.00	0.00	0.05	-0.02	-0.01	0.03	0.06	-0.04	0.00	0.03	0.15	0.07	0.07
Health problem: anaemia	0.05	-0.01	-0.03	0.02	0.00	0.00	0.00	0.00	0.00	0.00	0.00	-0.08	-0.01	-0.03	-0.05	0.02	0.03	0.08	0.07	0.09	0.08
Health problem: diabetes	0.05	0.03	0.02	0.03	0.00	0.00	0.00	0.00	0.00	0.00	0.06	0.00	-0.01	0.00	0.06	-0.07	0.00	0.03	0.12	0.12	0.10
Health problem: cancer	-0.02	-0.02	0.01	0.04	0.00	0.00	0.00	0.00	0.00	0.00	0.04	-0.02	0.07	0.02	0.02	-0.02	0.02	0.02	0.06	0.07	0.04
Health problem: liver	-0.01	0.06	0.02	0.05	0.00	0.00	0.00	0.00	0.00	0.00	0.04	0.02	-0.01	0.03	0.03	-0.02	0.02	0.02	0.08	0.05	0.05
Vaccine: mRNA-1273	-0.04	0.03	-0.01	0.03	0.00	0.00	0.00	0.00	0.00	0.00	0.02	-0.03	-0.02	0.04	-0.03	0.04	-0.02	-0.03	-0.03	0.02	0.03
Anxiety or depression : PHQ-4	0.04	0.24	0.15	0.09	0.00	0.00	0.00	0.00	0.00	0.00	-0.19	-0.14	0.12	0.01	0.06	-0.04	0.03	0.03	0.12	0.42	0.16
Somatosensory amplification	0.08	0.12	0.08	0.06	0.00	0.00	0.00	0.00	0.00	0.00	-0.11	-0.33	0.03	0.00	-0.01	0.03	0.05	-0.04	0.14	0.18	0.17
Benefit of vaccination	-0.01	-0.03	-0.04	-0.10	0.00	0.00	0.00	0.00	0.00	0.00	0.06	-0.04	0.02	-0.04	-0.03	0.06	-0.02	-0.02	-0.08	-0.04	-0.08
Risk for COVID-19 infection	0.00	0.01	0.00	-0.06	0.00	0.00	0.00	0.00	0.00	0.00	0.06	-0.04	0.02	-0.02	-0.04	0.05	-0.04	0.05	-0.02	-0.01	-0.01
Risk for hospitalisation (COVID-19)	0.01	-0.01	0.00	0.00	0.00	0.00	0.00	0.00	0.00	0.00	0.23	-0.07	0.01	0.04	0.03	-0.06	-0.06	0.09	0.08	0.01	0.06
Risk for side effects	0.06	0.11	0.06	0.12	0.00	0.00	0.00	0.00	0.00	0.00	-0.13	-0.14	0.01	-0.03	-0.04	0.06	0.02	0.02	0.04	0.03	0.09
Risk for hospitalisation (vaccination)	0.02	0.03	0.01	0.13	0.00	0.00	0.00	0.00	0.00	0.00	0.00	-0.06	0.02	0.04	0.10	-0.16	0.02	0.17	0.14	0.06	0.09
Risk for long-term side effects	0.00	0.02	0.06	0.13	0.00	0.00	0.00	0.00	0.00	0.00	0.05	-0.10	0.03	0.06	0.09	-0.13	0.04	0.13	0.15	0.03	0.12
Side effects at first vaccination	0.00	0.13	0.13	0.15	0.00	0.00	0.00	0.00	0.00	0.00	-0.12	-0.12	-0.01	0.01	0.03	-0.02	0.00	0.07	0.04	0.01	0.06
Side effects observed in others	0.00	0.08	0.07	0.08	0.00	0.00	0.00	0.00	0.00	0.00	-0.12	-0.11	0.04	-0.03	0.00	0.02	0.03	0.00	0.05	0.05	0.10

	Health problem: hypertension	Health problem: pulmonary	Health problem: rheumatism	Health problem: heart	Health problem: osteoarthritis	Health problem: renal disease	Health problem: anaemia	Health problem: diabetes	Health problem: cancer	Health problem: liver	Vaccine: mRNA-1273	Anxiety or depression : PHQ-4	Somatosensory amplification	Benefit of vaccination	Risk for COVID-19 infection	Risk for hospitalisation (COVID-19)	Risk for systemic side effects	Risk for hospitalisation (vaccination)	Risk for long-term side effects	Side effects at first vaccination	Side effects observed in others
Health problem: hypertension	1.00																				
Health problem: pulmonary	0.00	1.00																			
Health problem: rheumatism	0.07	0.10	1.00																		
Health problem: heart	0.30	0.08	0.03	1.00																	
Health problem: osteoarthritis	0.13	0.10	0.21	0.06	1.00																
Health problem: renal disease	0.14	0.06	0.07	0.10	0.12	1.00															
Health problem: anaemia	-0.01	0.06	0.06	0.05	0.06	0.01	1.00														
Health problem: diabetes	0.16	0.07	0.08	0.11	0.11	0.18	0.04	1.00													
Health problem: cancer	0.12	0.02	0.03	0.08	0.13	0.18	0.06	0.20	1.00												
Health problem: liver	0.10	0.03	0.07	0.02	0.12	0.12	0.03	-0.01	0.05	1.00											
Vaccine: mRNA-1273	-0.01	-0.03	0.03	-0.01	0.03	0.03	0.05	0.01	0.01	-0.02	1.00										
Anxiety or depression : PHQ-4	0.00	0.04	0.02	0.01	0.05	0.05	0.05	0.08	0.01	0.04	-0.02	1.00									
Somatosensory amplification	0.02	0.10	0.04	0.02	0.01	0.03	0.10	0.01	0.02	-0.03	0.02	0.28	1.00								
Benefit of vaccination	0.01	-0.03	-0.03	-0.03	-0.02	-0.04	0.01	-0.06	-0.01	-0.06	0.11	-0.09	-0.06	1.00							
Risk for COVID-19 infection	0.02	0.00	-0.01	0.06	-0.01	-0.02	-0.03	-0.02	-0.01	-0.03	0.02	-0.01	0.03	0.27	1.00						
Risk for hospitalisation (COVID-19)	0.11	0.07	0.03	0.11	0.06	0.01	0.03	0.08	0.05	-0.01	0.01	-0.03	0.01	0.26	0.51	1.00					
Risk for side effects	0.00	0.07	0.02	0.02	-0.01	0.01	0.04	0.02	0.00	-0.03	0.07	0.14	0.23	0.00	0.18	0.10	1.00				
Risk for hospitalisation (vaccination)	0.06	0.07	0.03	0.07	0.03	0.04	0.02	0.09	0.00	0.03	-0.01	0.08	0.05	-0.15	0.11	0.28	0.31	1.00			
Risk for long-term side effects	0.06	0.09	0.04	0.06	0.04	0.04	0.04	0.07	0.00	0.06	-0.05	0.09	0.11	-0.29	-0.02	0.14	0.22	0.61	1.00		
Side effects at first vaccination	-0.01	0.06	-0.02	0.05	-0.01	0.01	0.03	0.01	0.01	-0.02	0.09	0.16	0.18	-0.09	0.00	0.04	0.41	0.23	0.21	1.00	
Side effects observed in others	0.02	0.06	0.01	0.03	0.00	0.02	0.02	0.06	0.06	-0.01	-0.03	0.13	0.14	-0.08	-0.01	0.03	0.30	0.16	0.17	0.31	1.00

- r < |0.10|
- |0.10| ≤ r ≤ |0.19|
- |0.20| ≤ r ≤ |0.29|
- |0.30| ≤ r ≤ |0.39|
- |0.40| ≤ r ≤ |0.49|
- |0.50| ≤ r ≤ |0.59|
- |0.60| ≤ r ≤ |0.69|
- |0.70| ≤ r ≤ |0.79|
- |0.80| ≤ r ≤ |0.89|
- r ≥ |0.90|

© 2023 Schäfer I et al. *JAMA Network Open*.

eTable 1. Unadjusted Association Between Expected Benefit of Vaccination and Systemic Adverse Effects: Results of Mixed-Effects Ordered Logistic Regression Analysis Adjusted for Random Effects on the Patient Level

Characteristic	OR (95% CI)	p
Highest symptom level in the last two weeks before vaccination:		
- no symptoms	reference	
- light symptoms	2.05 (1.65/2.55)	<0.001
- moderate symptoms	3.54 (2.68/4.68)	<0.001
- severe symptoms	4.93 (2.97/8.18)	<0.001
- very severe symptoms	13.0 (5.57/30.5)	<0.001
Time of observation:		
- day one after vaccination	reference	
- day two after vaccination	4.99 (4.31/5.79)	<0.001
- day three after vaccination	0.54 (0.46/0.63)	<0.001
- day four after vaccination	0.19 (0.16/0.23)	<0.001
- day five after vaccination	0.09 (0.07/0.11)	<0.001
- day six after vaccination	0.05 (0.04/0.06)	<0.001
- day seven after vaccination	0.03 (0.02/0.04)	<0.001
Expected benefit of vaccination (per 3 points difference)	0.74 (0.65/0.86)	<0.001

OR: odds ratio; CI: confidence interval; n: number of participants (N = 1678); N: number of observations (N = 10 447)

eTable 2. Unadjusted Association Between Expected Risk for COVID-19 Infection and Systemic Adverse Effects: Results of Mixed-Effects Ordered Logistic Regression Analysis Adjusted for Random Effects on the Patient Level

Characteristic	OR (95% CI)	p
Highest symptom level in the last two weeks before vaccination:		
- no symptoms	reference	
- light symptoms	2.09 (1.69/2.60)	<0.001
- moderate symptoms	3.64 (2.75/4.82)	<0.001
- severe symptoms	5.23 (3.15/8.70)	<0.001
- very severe symptoms	15.4 (6.58/36.2)	<0.001
Time of observation:		
- day one after vaccination	reference	
- day two after vaccination	4.99 (4.30/5.78)	<0.001
- day three after vaccination	0.54 (0.46/0.63)	<0.001
- day four after vaccination	0.19 (0.16/0.22)	<0.001
- day five after vaccination	0.09 (0.07/0.11)	<0.001
- day six after vaccination	0.05 (0.04/0.06)	<0.001
- day seven after vaccination	0.03 (0.02/0.04)	<0.001
Expected risk for COVID-19 infection (per 3 points difference)	0.99 (0.89/1.10)	0.84

OR: odds ratio; CI: confidence interval; n: number of participants (n = 1678); N: number of observations (N = 10 447)

© 2023 Schäfer I et al. *JAMA Network Open*.

eTable 3. Unadjusted Association Between Expected Risk for Hospitalization due to COVID-19 Infection and Systemic Adverse Effects: Results of Mixed-Effects Ordered Logistic Regression Analysis Adjusted for Random Effects on the Patient Level

Characteristic	OR (95% CI)	p
Highest symptom level in the last two weeks before vaccination:		
- no symptoms	reference	
- light symptoms	2.10 (1.69/2.61)	<0.001
- moderate symptoms	3.64 (2.75/4.82)	<0.001
- severe symptoms	5.24 (3.15/8.71)	<0.001
- very severe symptoms	15.5 (6.61/36.2)	<0.001
Time of observation:		
- day one after vaccination	reference	
- day two after vaccination	4.99 (4.30/5.79)	<0.001
- day three after vaccination	0.54 (0.46/0.63)	<0.001
- day four after vaccination	0.19 (0.16/0.22)	<0.001
- day five after vaccination	0.09 (0.07/0.11)	<0.001
- day six after vaccination	0.05 (0.04/0.06)	<0.001
- day seven after vaccination	0.03 (0.02/0.04)	<0.001
Expected risk for hospitalisation due to COVID-19 infection (per 3 points difference)	0.88 (0.79/0.99)	0.03

OR: odds ratio; CI: confidence interval; n: number of participants (N = 1678); N: number of observations (N = 10 447)

eTable 4. Unadjusted Association Between Expected Risk for Adverse Effects of Vaccination and Systemic Adverse Effects: Results of Mixed-Effects Ordered Logistic Regression Analysis Adjusted for Random Effects on the Patient Level

Characteristic	OR (95% CI)	p
Highest symptom level in the last two weeks before vaccination:		
- no symptoms	reference	
- light symptoms	1.77 (1.44/2.19)	<0.001
- moderate symptoms	2.79 (2.12/3.68)	<0.001
- severe symptoms	4.02 (2.46/6.59)	<0.001
- very severe symptoms	7.77 (3.38/17.9)	<0.001
Time of observation:		
- day one after vaccination	reference	
- day two after vaccination	4.97 (4.29/5.77)	<0.001
- day three after vaccination	0.54 (0.46/0.63)	<0.001
- day four after vaccination	0.19 (0.16/0.22)	<0.001
- day five after vaccination	0.09 (0.07/0.11)	<0.001
- day six after vaccination	0.05 (0.04/0.06)	<0.001
- day seven after vaccination	0.03 (0.02/0.04)	<0.001
Expected risk for adverse effects of vaccination (per 3 points difference)	1.86 (1.66/2.10)	<0.001

OR: odds ratio; CI: confidence interval; n: number of participants (N = 1678); N: number of observations (N = 10 447)

© 2023 Schäfer I et al. *JAMA Network Open*.

eTable 5. Unadjusted Association Between Expected Risk for Hospitalization Due to Adverse Effects of Vaccination and Systemic Adverse Effects: Results of Mixed-Effects Ordered Logistic Regression Analysis Adjusted for Random Effects on the Patient Level

Characteristic	OR (95% CI)	p
Highest symptom level in the last two weeks before vaccination:		
- no symptoms	reference	
- light symptoms	2.07 (1.67/2.58)	<0.001
- moderate symptoms	3.59 (2.71/4.76)	<0.001
- severe symptoms	5.17 (3.11/8.59)	<0.001
- very severe symptoms	14.0 (5.95/33.2)	<0.001
Time of observation:		
- day one after vaccination	reference	
- day two after vaccination	4.98 (4.30/5.78)	<0.001
- day three after vaccination	0.54 (0.46/0.63)	<0.001
- day four after vaccination	0.19 (0.16/0.22)	<0.001
- day five after vaccination	0.09 (0.07/0.11)	<0.001
- day six after vaccination	0.05 (0.04/0.06)	<0.001
- day seven after vaccination	0.03 (0.02/0.04)	<0.001
Expected risk for hospitalisation due to adverse effects of vaccination (per 3 points difference)	1.13 (0.98/1.31)	0.10

OR: odds ratio; CI: confidence interval; n: number of participants (N = 1678); N: number of observations (N = 10 447)

eTable 6. Unadjusted Association Between Expected Risk for Long-term Adverse Effects of Vaccination and Systemic Adverse Effects: Results of Mixed-Effects Ordered Logistic Regression Analysis Adjusted for Random Effects on the Patient Level

Characteristic	OR (95% CI)	P
Highest symptom level in the last two weeks before vaccination:		
- no symptoms	reference	
- light symptoms	2.07 (1.67/2.57)	<0.001
- moderate symptoms	3.58 (2.71/4.74)	<0.001
- severe symptoms	5.01 (3.01/8.32)	<0.001
- very severe symptoms	13.5 (5.75/31.9)	<0.001
Time of observation:		
- day one after vaccination	reference	
- day two after vaccination	4.98 (4.29/5.77)	<0.001
- day three after vaccination	0.54 (0.46/0.63)	<0.001
- day four after vaccination	0.19 (0.16/0.22)	<0.001
- day five after vaccination	0.09 (0.07/0.11)	<0.001
- day six after vaccination	0.05 (0.04/0.06)	<0.001
- day seven after vaccination	0.03 (0.02/0.04)	<0.001
Expected risk for long-term adverse effects of vaccination (per 3 points difference)	1.16 (1.02/1.32)	0.03

OR: odds ratio; CI: confidence interval; n: number of participants (N = 1678); N: number of observations (N = 10 447)

© 2023 Schäfer I et al. *JAMA Network Open*.

eTable 7. Unadjusted Association Between Adverse Effects Experienced at First Vaccination and Systemic Adverse Effects: Results of Mixed-Effects Ordered Logistic Regression Analysis Adjusted for Random Effects on the Patient Level

**Characteristic**	**OR (95% CI)**	**p**
Highest symptom level in the last two weeks before vaccination:		
- no symptoms	reference	
- light symptoms	1.81 (1.47/2.23)	<0.001
- moderate symptoms	2.65 (2.02/3.48)	<0.001
- severe symptoms	3.18 (1.95/5.20)	<0.001
- very severe symptoms	6.00 (2.63/13.7)	<0.001
Time of observation:		
- day one after vaccination	reference	
- day two after vaccination	4.98 (4.30/5.77)	<0.001
- day three after vaccination	0.54 (0.46/0.63)	<0.001
- day four after vaccination	0.19 (0.16/0.22)	<0.001
- day five after vaccination	0.09 (0.07/0.11)	<0.001
- day six after vaccination	0.05 (0.04/0.06)	<0.001
- day seven after vaccination	0.03 (0.02/0.04)	<0.001
Adverse effects experienced at first vaccination (per 3 points difference)	2.07 (1.84/2.34)	<0.001

OR: odds ratio; CI: confidence interval; n: number of participants (N = 1678); N: number of observations (N = 10 447)

eTable 8. Unadjusted Association Between Adverse Effects Observed in Close Contacts and Systemic Adverse Effects: Results of Mixed-Effects Ordered Logistic Regression Analysis Adjusted for Random Effects on the Patient Level

**Characteristic**	**OR (95% CI)**	**p**
Highest symptom level in the last two weeks before vaccination:		
- no symptoms	reference	
- light symptoms	2.02 (1.63/2.50)	<0.001
- moderate symptoms	3.32 (2.52/4.39)	<0.001
- severe symptoms	4.55 (2.75/7.54)	<0.001
- very severe symptoms	11.9 (5.12/27.7)	<0.001
Time of observation:		
- day one after vaccination	reference	
- day two after vaccination	4.98 (4.29/5.77)	<0.001
- day three after vaccination	0.54 (0.46/0.63)	<0.001
- day four after vaccination	0.19 (0.16/0.22)	<0.001
- day five after vaccination	0.09 (0.07/0.11)	<0.001
- day six after vaccination	0.05 (0.04/0.06)	<0.001
- day seven after vaccination	0.03 (0.02/0.04)	<0.001
Adverse effects observed in close contacts (per 3 points difference)	1.45 (1.28/1.64)	<0.001

OR: odds ratio; CI: confidence interval; n: number of participants (N = 1678); N: number of observations (N = 10 447)

© 2023 Schäfer I et al. *JAMA Network Open*.

eTable 9. Unadjusted Association Between Anxiety and Depression and Systemic Adverse Effects: Results of Mixed-Effects Ordered Logistic Regression Analysis Adjusted for Random Effects on the Patient Level

Characteristic	OR (95% CI)	p
Highest symptom level in the last two weeks before vaccination:		
- no symptoms	reference	
- light symptoms	1.81 (1.46/2.26)	<0.001
- moderate symptoms	2.71 (2.02/3.62)	<0.001
- severe symptoms	3.68 (2.20/6.15)	<0.001
- very severe symptoms	10.5 (4.48/24.4)	<0.001
Time of observation:		
- day one after vaccination	reference	
- day two after vaccination	4.98 (4.29/5.77)	<0.001
- day three after vaccination	0.54 (0.46/0.63)	<0.001
- day four after vaccination	0.19 (0.16/0.23)	<0.001
- day five after vaccination	0.09 (0.07/0.11)	<0.001
- day six after vaccination	0.05 (0.04/0.06)	<0.001
- day seven after vaccination	0.03 (0.02/0.04)	<0.001
Anxiety or depression (pursuant to PHQ-4, per 2 points difference)	1.33 (1.21/1.46)	<0.001

OR: odds ratio; CI: confidence interval; n: number of participants (N = 1678); N: number of observations (N = 10 447)

eTable 10. Unadjusted Association Between Somatosensory Amplification and Systemic Adverse Effects: Results of Mixed-Effects Ordered Logistic Regression Analysis Adjusted for Random Effects on the Patient Level

Characteristic	OR (95% CI)	p
Highest symptom level in the last two weeks before vaccination:		
- no symptoms	reference	
- light symptoms	1.79 (1.45/2.22)	<0.001
- moderate symptoms	2.90 (2.20/3.84)	<0.001
- severe symptoms	4.02 (2.44/6.63)	<0.001
- very severe symptoms	11.2 (4.86/25.8)	<0.001
Time of observation:		
- day one after vaccination	reference	
- day two after vaccination	4.99 (4.31/5.79)	<0.001
- day three after vaccination	0.54 (0.46/0.63)	<0.001
- day four after vaccination	0.19 (0.16/0.22)	<0.001
- day five after vaccination	0.09 (0.07/0.11)	<0.001
- day six after vaccination	0.05 (0.04/0.06)	<0.001
- day seven after vaccination	0.03 (0.02/0.04)	<0.001
Somatosensory Amplification (per 8 points difference)	1.75 (1.53/1.99)	<0.001

OR: odds ratio; CI: confidence interval; n: number of participants (N = 1678); N: number of observations (N = 10 447)

© 2023 Schäfer I et al. *JAMA Network Open*.

eTable 11. Associations With Tiredness/Fatigue: Results of Mixed-Effects Ordered Logistic Regression Analysis Adjusted for Random Effects on the Patient Level

**Characteristic**	**OR (95% CI)**	**p**
Highest symptom level in the last two weeks before vaccination:		
- no symptoms	reference	
- light symptoms	1.68 (1.38/2.05)	<0.001
- moderate symptoms	2.04 (1.50/2.79)	<0.001
- severe symptoms	3.00 (1.65/5.46)	<0.001
- very severe symptoms	8.28 (2.39/28.7)	0.001
Time of observation:		
- day one after vaccination	reference	
- day two after vaccination	4.71 (4.05/5.49)	<0.001
- day three after vaccination	0.50 (0.43/0.59)	<0.001
- day four after vaccination	0.18 (0.15/0.21)	<0.001
- day five after vaccination	0.08 (0.06/0.10)	<0.001
- day six after vaccination	0.05 (0.04/0.06)	<0.001
- day seven after vaccination	0.02 (0.02/0.03)	<0.001
Age (per 10 years)	0.98 (0.90/1.06)	0.56
Sex:		
- female	reference	
- male	0.70 (0.58/0.85)	<0.001
- non-binary	1.41 (0.41/4.77)	0.58
Living arrangement:		
- living together with others	reference	
- living alone	1.04 (0.85/1.27)	0.68
Educational level (pursuant to CASMIN):		
- higher or lower tertiary education	reference	
- secondary school certificate or “A” level equivalent	0.86 (0.71/1.04)	0.11
- inadequately completed, general elementary or basic vocational	0.48 (0.30/0.78)	0.003
Migration status:		
- participant and both parents born in Germany	reference	
- participant born in Germany and at least one parent born abroad	0.94 (0.73/1.22)	0.65
- participant born abroad	0.82 (0.62/1.07)	0.15

OR: odds ratio; CI: confidence interval; n: number of participants (N = 1678); N: number of observations (N = 10 447); CASMIN: Comparative Analysis of Social Mobility in Industrial Nations

© 2023 Schäfer I et al. *JAMA Network Open*.

eTable 11. Associations With Tiredness/Fatigue: Results of Mixed-Effects Ordered Logistic Regression Analysis Adjusted for Random Effects on the Patient Level [continued]

**Characteristic**	**OR (95% CI)**	**p**
Self-reported health problems:		
- Back pain	1.29 (1.03/1.60)	0.03
- Depression	1.31 (0.99/1.73)	0.06
- Gastrointestinal complaints	1.48 (1.10/1.99)	0.009
- Hypertension	0.73 (0.50/1.08)	0.11
- Pulmonary disease	1.32 (0.90/1.94)	0.16
- Rheumatism or other autoimmune disease	0.76 (0.50/1.16)	0.21
- Heart disease	1.03 (0.60/1.80)	0.91
- Osteoarthritis	0.90 (0.54/1.51)	0.69
- Renal disease	0.79 (0.41/1.52)	0.48
- Anaemia or other blood disease	1.22 (0.67/2.24)	0.51
- Diabetes mellitus	0.49 (0.24/1.01)	0.05
- Cancer	0.90 (0.39/2.09)	0.80
- Liver disease	0.79 (0.30/2.12)	0.64
Anxiety or depression (pursuant to PHQ-4, per 2 points difference)	1.13 (1.02/1.25)	0.01
Vaccine:		
- BNT162b2	reference	
- mRNA-1273	2.41 (1.96/2.97)	<0.001
Somatosensory Amplification (per 8 points difference)	1.20 (1.04/1.37)	0.01
Expected benefit of vaccination (per 3 points difference)	0.79 (0.68/0.91)	0.001
Expected risk for COVID-19 infection (per 3 points difference)	1.02 (0.91/1.15)	0.73
Expected risk for hospitalisation due to COVID-19 infection (per 3 points difference)	0.87 (0.77/0.99)	0.04
Expected risk for adverse effects of vaccination (per 3 points difference)	1.39 (1.22/1.58)	<0.001
Expected risk for hospitalisation due to adverse effects of vaccination (per 3 points difference)	0.81 (0.67/0.97)	0.02
Expected risk for long-term adverse effects of vaccination (per 3 points difference)	1.01 (0.86/1.18)	0.94
Adverse effects experienced at first vaccination (per 3 points difference)	1.55 (1.36/1.77)	<0.001
Adverse effects observed in close contacts (per 3 points difference)	1.15 (1.01/1.30)	0.03

OR: odds ratio; CI: confidence interval; n: number of participants (n = 1678); N: number of observations (N = 10 447); PHQ: patient health questionnaire

© 2023 Schäfer I et al. *JAMA Network Open*.

eTable 12. Associations With Headache: Results of Mixed-Effects Ordered Logistic Regression Analysis Adjusted for Random Effects on the Patient Level

Characteristic	OR (95% CI)	p
Highest symptom level in the last two weeks before vaccination:		
- no symptoms	reference	
- light symptoms	1.92 (1.53/2.41)	<0.001
- moderate symptoms	2.20 (1.51/3.22)	<0.001
- severe symptoms	3.52 (1.68/7.38)	0.001
- very severe symptoms	5.22 (0.91/29.8)	0.06
Time of observation:		
- day one after vaccination	reference	
- day two after vaccination	5.71 (4.82/6.77)	<0.001
- day three after vaccination	0.85 (0.71/1.02)	0.08
- day four after vaccination	0.29 (0.24/0.36)	<0.001
- day five after vaccination	0.14 (0.11/0.17)	<0.001
- day six after vaccination	0.08 (0.06/0.10)	<0.001
- day seven after vaccination	0.01 (0.01/0.02)	<0.001
Age (per 10 years)	0.88 (0.80/0.97)	0.009
Sex:		
- female	reference	
- male	0.78 (0.63/0.96)	0.02
- non-binary	0.99 (0.26/3.85)	0.99
Living arrangement:		
- living together with others	reference	
- living alone	0.94 (0.76/1.18)	0.60
Educational level (pursuant to CASMIN):		
- higher or lower tertiary education	reference	
- secondary school certificate or "A" level equivalent	1.03 (0.84/1.26)	0.80
- inadequately completed, general elementary or basic vocational	0.73 (0.44/1.23)	0.24
Migration status:		
- participant and both parents born in Germany	reference	
- participant born in Germany and at least one parent born abroad	1.01 (0.77/1.34)	0.92
- participant born abroad	0.85 (0.63/1.14)	0.28

OR: odds ratio; CI: confidence interval; n: number of participants (n = 1678); N: number of observations (N = 10 447); CASMIN: Comparative Analysis of Social Mobility in Industrial Nations

© 2023 Schäfer I et al. *JAMA Network Open*.

eTable 12. Associations With Headache: Results of Mixed-Effects Ordered Logistic Regression Analysis Adjusted for Random Effects on the Patient Level [continued]

**Characteristic**	**OR (95% CI)**	**P**
Self-reported health problems:		
- Back pain	1.49 (1.17/1.90)	0.001
- Depression	1.26 (0.93/1.72)	0.14
- Gastrointestinal complaints	1.23 (0.89/1.70)	0.21
- Hypertension	0.69 (0.44/1.06)	0.09
- Pulmonary disease	1.29 (0.85/1.97)	0.23
- Rheumatism or other autoimmune disease	0.77 (0.48/1.22)	0.27
- Heart disease	0.60 (0.31/1.15)	0.12
- Osteoarthritis	1.08 (0.62/1.89)	0.78
- Renal disease	0.81 (0.40/1.67)	0.57
- Anaemia or other blood disease	1.19 (0.61/2.30)	0.61
- Diabetes mellitus	0.65 (0.30/1.42)	0.28
- Cancer	1.44 (0.57/3.64)	0.44
- Liver disease	0.91 (0.31/2.66)	0.87
Anxiety or depression (pursuant to PHQ-4, per 2 points difference)	1.12 (1.01/1.24)	0.04
Vaccine:		
- BNT162b2	reference	
- mRNA-1273	2.73 (2.18/3.42)	<0.001
Somatosensory Amplification (per 8 points difference)	1.26 (1.09/1.47)	0.002
Expected benefit of vaccination (per 3 points difference)	0.80 (0.68/0.95)	0.008
Expected risk for COVID-19 infection (per 3 points difference)	0.98 (0.86/1.11)	0.74
Expected risk for hospitalisation due to COVID-19 infection (per 3 points difference)	0.89 (0.77/1.02)	0.10
Expected risk for adverse effects of vaccination (per 3 points difference)	1.36 (1.17/1.57)	<0.001
Expected risk for hospitalisation due to adverse effects of vaccination (per 3 points difference)	0.92 (0.76/1.12)	0.42
Expected risk for long-term adverse effects of vaccination (per 3 points difference)	0.95 (0.80/1.13)	0.59
Adverse effects experienced at first vaccination (per 3 points difference)	1.48 (1.29/1.71)	<0.001
Adverse effects observed in close contacts (per 3 points difference)	1.11 (0.97/1.27)	0.14

OR: odds ratio; CI: confidence interval; n: number of participants (n = 1678); N: number of observations (N = 10 447); PHQ: patient health questionnaire

© 2023 Schäfer I et al. *JAMA Network Open*.

eTable 13. Associations With Aching Limbs: Results of Mixed-Effects Ordered Logistic Regression Analysis Adjusted for Random Effects on the Patient Level

Characteristic	OR (95% CI)	p
Highest symptom level in the last two weeks before vaccination:		
- no symptoms	reference	
- light symptoms	2.37 (1.67/3.37)	<0.001
- moderate symptoms	2.74 (1.47/5.12)	0.002
- severe symptoms	2.78 (0.92/8.33)	0.07
- very severe symptoms	70.2 (11.3/438)	<0.001
Time of observation:		
- day one after vaccination	reference	
- day two after vaccination	5.32 (4.43/6.38)	<0.001
- day three after vaccination	0.56 (0.46/0.69)	<0.001
- day four after vaccination	0.17 (0.13/0.22)	<0.001
- day five after vaccination	0.06 (0.04/0.08)	<0.001
- day six after vaccination	0.03 (0.02/0.05)	<0.001
- day seven after vaccination	0.02 (0.01/0.03)	<0.001
Age (per 10 years)	0.90 (0.81/1.00)	0.05
Sex:		
- female	reference	
- male	1.00 (0.78/1.27)	0.97
- non-binary	0.23 (0.04/1.34)	0.10
Living arrangement:		
- living together with others	reference	
- living alone	1.06 (0.82/1.37)	0.64
Educational level (pursuant to CASMIN):		
- higher or lower tertiary education	reference	
- secondary school certificate or "A" level equivalent	1.19 (0.81/1.54)	0.16
- inadequately completed, general elementary or basic vocational	0.76 (0.42/1.38)	0.37
Migration status:		
- participant and both parents born in Germany	reference	
- participant born in Germany and at least one parent born abroad	1.11 (0.81/1.54)	0.52
- participant born abroad	0.79 (0.56/1.12)	0.19

OR: odds ratio; CI: confidence interval; n: number of participants (n = 1678); N: number of observations (N = 10 447); CASMIN: Comparative Analysis of Social Mobility in Industrial Nations

© 2023 Schäfer I et al. *JAMA Network Open*.

eTable 13. Associations With Aching Limbs: Results of Mixed-Effects Ordered Logistic Regression Analysis Adjusted for Random Effects on the Patient Level [continued]

Characteristic	OR (95% CI)	p
Self-reported health problems:		
- Back pain	1.47 (1.11/1.94)	0.006
- Depression	1.43 (1.00/2.04)	0.05
- Gastrointestinal complaints	1.42 (0.98/2.06)	0.07
- Hypertension	0.59 (0.36/0.96)	0.04
- Pulmonary disease	1.92 (1.19/3.09)	0.007
- Rheumatism or other autoimmune disease	0.91 (0.53/1.56)	0.73
- Heart disease	1.46 (0.74/2.90)	0.28
- Osteoarthritis	0.59 (0.30/1.15)	0.12
- Renal disease	0.63 (0.27/1.47)	0.28
- Anaemia or other blood disease	1.47 (0.70/3.10)	0.31
- Diabetes mellitus	0.41 (0.16/1.05)	0.06
- Cancer	1.77 (0.62/5.06)	0.29
- Liver disease	1.21 (0.36/4.14)	0.76
Anxiety or depression (pursuant to PHQ-4, per 2 points difference)	1.17 (1.04/1.32)	0.008
Vaccine:		
- BNT162b2	reference	
- mRNA-1273	3.34 (2.57/4.34)	<0.001
Somatosensory Amplification (per 8 points difference)	1.19 (1.00/1.41)	0.05
Expected benefit of vaccination (per 3 points difference)	0.77 (0.64/0.93)	0.007
Expected risk for COVID-19 infection (per 3 points difference)	1.00 (0.86/1.16)	0.99
Expected risk for hospitalisation due to COVID-19 infection (per 3 points difference)	0.90 (0.76/1.07)	0.23
Expected risk for adverse effects of vaccination (per 3 points difference)	1.38 (1.17/1.63)	<0.001
Expected risk for hospitalisation due to adverse effects of vaccination (per 3 points difference)	1.00 (0.79/1.26)	0.97
Expected risk for long-term adverse effects of vaccination (per 3 points difference)	0.89 (0.73/1.09)	0.27
Adverse effects experienced at first vaccination (per 3 points difference)	1.25 (1.06/1.47)	0.008
Adverse effects observed in close contacts (per 3 points difference)	1.16 (0.99/1.36)	0.06

OR: odds ratio; CI: confidence interval; n: number of participants (n = 1678)**; N: number of observations** (N = 10 447)**; PHQ: patient health questionnaire**

© 2023 Schäfer I et al. *JAMA Network Open*.

eTable 14. Associations With Joint Pain: Results of Mixed-Effects Ordered Logistic Regression Analysis Adjusted for Random Effects on the Patient Level

Characteristic	OR (95% CI)	p
Highest symptom level in the last two weeks before vaccination:		
- no symptoms	reference	
- light symptoms	3.16 (2.27/4.40)	<0.001
- moderate symptoms	2.87 (1.53/5.37)	0.001
- severe symptoms	2.76 (0.68/11.1)	0.16
- very severe symptoms	11.3 (1.81/70.0)	0.009
Time of observation:		
- day one after vaccination	reference	
- day two after vaccination	5.17 (4.23/6.32)	<0.001
- day three after vaccination	0.61 (0.48/0.77)	<0.001
- day four after vaccination	0.19 (0.14/0.25)	<0.001
- day five after vaccination	0.09 (0.06/0.13)	<0.001
- day six after vaccination	0.06 (0.04/0.09)	<0.001
- day seven after vaccination	0.17 (0.13/0.24)	<0.001
Age (per 10 years)	0.96 (0.86/1.07)	0.44
Sex:		
- female	reference	
- male	0.82 (0.64/1.06)	0.12
- non-binary	1.52 (0.36/6.52)	0.57
Living arrangement:		
- living together with others	reference	
- living alone	0.89 (0.68/1.16)	0.38
Educational level (pursuant to CASMIN):		
- higher or lower tertiary education	reference	
- secondary school certificate or “A” level equivalent	0.18 (0.93/1.53)	0.16
- inadequately completed, general elementary or basic vocational	0.85 (0.47/1.56)	0.61
Migration status:		
- participant and both parents born in Germany	reference	
- participant born in Germany and at least one parent born abroad	1.27 (0.91/1.77)	0.16
- participant born abroad	1.09 (0.77/1.55)	0.61

OR: odds ratio; CI: confidence interval; n: number of participants (n = 1678); N: number of observations (N = 10 447); CASMIN: Comparative Analysis of Social Mobility in Industrial Nations

© 2023 Schäfer I et al. *JAMA Network Open*.

eTable 14. Associations With Joint Pain: Results of Mixed-Effects Ordered Logistic Regression Analysis Adjusted for Random Effects on the Patient Level [continued]

**Characteristic**	**OR (95% CI)**	**p**
Self-reported health problems:		
- Back pain	1.58 (1.19/2.10)	0.002
- Depression	1.71 (1.19/2.45)	0.004
- Gastrointestinal complaints	1.37 (0.94/2.02)	0.11
- Hypertension	0.87 (0.53/1.43)	0.59
- Pulmonary disease	1.50 (0.92/2.46)	0.11
- Rheumatism or other autoimmune disease	0.89 (0.51/1.54)	0.67
- Heart disease	1.41 (0.71/2.80)	0.33
- Osteoarthritis	0.57 (0.28/1.14)	0.11
- Renal disease	0.56 (0.23/1.33)	0.19
- Anaemia or other blood disease	0.88 (0.40/1.92)	0.74
- Diabetes mellitus	0.35 (0.13/0.91)	0.03
- Cancer	1.96 (0.68/5.64)	0.21
- Liver disease	1.16 (0.33/4.00)	0.82
Anxiety or depression (pursuant to PHQ-4, per 2 points difference)	1.12 (0.99/1.26)	0.08
Vaccine:		
- BNT162b2	reference	
- mRNA-1273	3.02 (2.31/3.95)	<0.001
Somatosensory Amplification (per 8 points difference)	1.07 (0.90/1.28)	0.43
Expected benefit of vaccination (per 3 points difference)	0.77 (0.63/0.93)	0.008
Expected risk for COVID-19 infection (per 3 points difference)	0.89 (0.76/1.03)	0.13
Expected risk for hospitalisation due to COVID-19 infection (per 3 points difference)	0.98 (0.82/1.16)	0.80
Expected risk for adverse effects of vaccination (per 3 points difference)	1.24 (1.04/1.47)	0.01
Expected risk for hospitalisation due to adverse effects of vaccination (per 3 points difference)	1.09 (0.86/1.38)	0.48
Expected risk for long-term adverse effects of vaccination (per 3 points difference)	1.04 (0.84/1.27)	0.74
Adverse effects experienced at first vaccination (per 3 points difference)	1.36 (1.15/1.61)	<0.001
Adverse effects observed in close contacts (per 3 points difference)	1.10 (0.94/1.29)	0.24

OR: odds ratio; CI: confidence interval; n: number of participants (n = 1678)**; N: number of observations** (N = 10 447)**; PHQ: patient health questionnaire**

© 2023 Schäfer I et al. *JAMA Network Open*.

eTable 15. Associations With Chills: Results of Mixed-Effects Ordered Logistic Regression Analysis Adjusted for Random Effects on the Patient Level

**Characteristic**	**OR (95% CI)**	**p**
Highest symptom level in the last two weeks before vaccination:		
- no symptoms	reference	
- light symptoms	2.43 (1.53/3.86)	<0.001
- moderate symptoms	1.94 (0.79/4.76)	0.15
- severe symptoms	7.40 (2.45/22.4)	<0.001
- very severe symptoms	3.45 (0.14/84.3)	0.45
Time of observation:		
- day one after vaccination	reference	
- day two after vaccination	4.73 (3.86/5.81)	<0.001
- day three after vaccination	0.42 (0.32/0.54)	<0.001
- day four after vaccination	0.13 (0.09/0.19)	<0.001
- day five after vaccination	0.06 (0.04/0.10)	<0.001
- day six after vaccination	0.06 (0.04/0.09)	<0.001
- day seven after vaccination	0.02 (0.01/0.04)	<0.001
Age (per 10 years)	0.85 (0.77/0.94)	0.002
Sex:		
- female	reference	
- male	0.70 (0.55/0.89)	0.003
- non-binary	1.74 (0.45/6.67)	0.42
Living arrangement:		
- living together with others	reference	
- living alone	1.02 (0.80/1.30)	0.89
Educational level (pursuant to CASMIN):		
- higher or lower tertiary education	reference	
- secondary school certificate or “A” level equivalent	0.87 (0.69/1.10)	0.24
- inadequately completed, general elementary or basic vocational	0.38 (0.20/0.72)	0.003
Migration status:		
- participant and both parents born in Germany	reference	
- participant born in Germany and at least one parent born abroad	1.09 (0.80/1.49)	0.59
- participant born abroad	1.21 (0.88/1.67)	0.24

OR: odds ratio; CI: confidence interval; n: number of participants (n = 1678); N: number of observations (N = 10 447); **CASMIN: Comparative Analysis of Social Mobility in Industrial Nations**

© 2023 Schäfer I et al. *JAMA Network Open*.

eTable 15. Associations With Chills: Results of Mixed-Effects Ordered Logistic Regression Analysis Adjusted for Random Effects on the Patient Level [continued]

Characteristic	OR (95% CI)	P
Self-reported health problems:		
- Back pain	1.20 (0.91/1.57)	0.20
- Depression	1.08 (0.76/1.51)	0.68
- Gastrointestinal complaints	1.35 (0.95/1.92)	0.10
- Hypertension	0.73 (0.45/1.19)	0.21
- Pulmonary disease	0.94 (0.58/1.53)	0.82
- Rheumatism or other autoimmune disease	1.30 (0.79/2.14)	0.30
- Heart disease	1.19 (0.61/2.33)	0.61
- Osteoarthritis	0.58 (0.29/1.14)	0.12
- Renal disease	0.77 (0.34/1.71)	0.52
- Anaemia or other blood disease	1.85 (0.95/3.61)	0.07
- Diabetes mellitus	0.93 (0.39/2.18)	0.86
- Cancer	1.31 (0.49/3.54)	0.59
- Liver disease	1.41 (0.45/4.46)	0.56
Anxiety or depression (pursuant to PHQ-4, per 2 points difference)	1.14 (1.02/1.28)	0.02
Vaccine:		
- BNT162b2	reference	
- mRNA-1273	3.76 (2.94/4.81)	<0.001
Somatosensory Amplification (per 8 points difference)	1.05 (0.89/1.24)	0.54
Expected benefit of vaccination (per 3 points difference)	0.72 (0.61/0.87)	<0.001
Expected risk for COVID-19 infection (per 3 points difference)	0.95 (0.82/1.10)	0.48
Expected risk for hospitalisation due to COVID-19 infection (per 3 points difference)	1.11 (0.94/1.30)	0.22
Expected risk for adverse effects of vaccination (per 3 points difference)	1.17 (0.99/1.37)	0.06
Expected risk for hospitalisation due to adverse effects of vaccination (per 3 points difference)	1.00 (0.80/1.25)	0.99
Expected risk for long-term adverse effects of vaccination (per 3 points difference)	0.95 (0.78/1.15)	0.59
Adverse effects experienced at first vaccination (per 3 points difference)	1.38 (1.19/1.61)	<0.001
Adverse effects observed in close contacts (per 3 points difference)	1.06 (0.91/1.23)	0.44

OR: odds ratio; CI: confidence interval; n: number of participants (n = 1678); N: number of observations (N = 10 447); PHQ: patient health questionnaire

eTable 16. Associations With Fever: Results of Mixed-Effects Ordered Logistic Regression Analysis Adjusted for Random Effects on the Patient Level

**Characteristic**	**OR (95% CI)**	**p**
Highest symptom level in the last two weeks before vaccination:		
- no symptoms	reference	
- light symptoms	2.59 (1.24/5.43)	0.01
- moderate symptoms	22.9 (7.71/68.0)	<0.001
- severe symptoms	2.85 (0.23/35.3)	0.42
- very severe symptoms	23.0 (1.62/326)	0.02
Time of observation:		
- day one after vaccination	reference	
- day two after vaccination	5.65 (4.40/7.25)	<0.001
- day three after vaccination	0.54 (0.40/0.74)	<0.001
- day four after vaccination	0.12 (0.07/0.18)	<0.001
- day five after vaccination	0.08 (0.04/0.13)	<0.001
- day six after vaccination	0.03 (0.02/0.07)	<0.001
- day seven after vaccination	0.08 (0.04/0.14)	<0.001
Age (per 10 years)	0.93 (0.82/1.06)	0.28
Sex:		
- female	reference	
- male	0.72 (0.54/0.95)	0.02
- non-binary	0.79 (0.12/5.08)	0.81
Living arrangement:		
- living together with others	reference	
- living alone	0.83 (0.61/1.12)	0.22
Educational level (pursuant to CASMIN):		
- higher or lower tertiary education	reference	
- secondary school certificate or “A” level equivalent	0.91 (0.69/1.21)	0.52
- inadequately completed, general elementary or basic vocational	0.33 (0.15/0.75)	0.007
Migration status:		
- participant and both parents born in Germany	reference	
- participant born in Germany and at least one parent born abroad	1.36 (0.94/1.97)	0.10
- participant born abroad	0.94 (0.63/1.39)	0.74

OR: odds ratio; CI: confidence interval; n: number of participants (n = 1678); N: number of observations (N = 10 447); CASMIN: Comparative Analysis of Social Mobility in Industrial Nations

© 2023 Schäfer I et al. *JAMA Network Open*.

eTable 16. Associations With Fever: Results of Mixed-Effects Ordered Logistic Regression Analysis Adjusted for Random Effects on the Patient Level [continued]

Characteristic	OR (95% CI)	p
Self-reported health problems:		
- Back pain	1.29 (0.93/1.79)	0.13
- Depression	1.31 (0.86/2.00)	0.20
- Gastrointestinal complaints	1.03 (0.67/1.58)	0.90
- Hypertension	0.82 (0.45/1.48)	0.50
- Pulmonary disease	0.99 (0.55/1.78)	0.97
- Rheumatism or other autoimmune disease	1.14 (0.62/2.10)	0.68
- Heart disease	0.69 (0.28/1.69)	0.42
- Osteoarthritis	0.67 (0.30/1.51)	0.33
- Renal disease	0.54 (0.20/1.49)	0.23
- Anaemia or other blood disease	1.61 (0.71/3.65)	0.25
- Diabetes mellitus	0.79 (0.26/2.34)	0.66
- Cancer	2.06 (0.65/6.51)	0.22
- Liver disease	1.26 (0.31/5.11)	0.75
Anxiety or depression (pursuant to PHQ-4, per 2 points difference)	0.99 (0.86/1.14)	0.89
Vaccine:		
- BNT162b2	reference	
- mRNA-1273	3.89 (2.90/5.23)	<0.001
Somatosensory Amplification (per 8 points difference)	0.97 (0.79/1.19)	0.79
Expected benefit of vaccination (per 3 points difference)	0.71 (0.57/0.88)	0.002
Expected risk for COVID-19 infection (per 3 points difference)	0.98 (0.82/1.16)	0.79
Expected risk for hospitalisation due to COVID-19 infection (per 3 points difference)	0.92 (0.76/1.12)	0.43
Expected risk for adverse effects of vaccination (per 3 points difference)	1.28 (1.05/1.56)	0.01
Expected risk for hospitalisation due to adverse effects of vaccination (per 3 points difference)	1.00 (0.77/1.31)	0.98
Expected risk for long-term adverse effects of vaccination (per 3 points difference)	0.92 (0.73/1.16)	0.46
Adverse effects experienced at first vaccination (per 3 points difference)	1.66 (1.38/1.99)	<0.001
Adverse effects observed in close contacts (per 3 points difference)	1.12 (0.93/1.35)	0.22

OR: odds ratio; CI: confidence interval; n: number of participants (n = 1678); N: number of observations (N = 10 447); **PHQ: patient health questionnaire**

© 2023 Schäfer I et al. *JAMA Network Open*.

eTable 17. Associations With Deep Leg Pain: Results of Mixed-Effects Ordered Logistic Regression Analysis Adjusted for Random Effects on the Patient Level

Characteristic	OR (95% CI)	p
Highest symptom level in the last two weeks before vaccination:		
- no symptoms	reference	
- light symptoms	4.42 (2.43/8.04)	<0.001
- moderate symptoms	3.94 (1.24/12.5)	0.02
- severe symptoms	73.7 (0.95/5725)	0.05
- very severe symptoms	54.3 (0.48/6083)	0.10
Time of observation:		
- day one after vaccination	reference	
- day two after vaccination	4.63 (3.50/6.13)	<0.001
- day three after vaccination	0.60 (0.43/0.84)	0.003
- day four after vaccination	0.20 (0.13/0.31)	<0.001
- day five after vaccination	0.13 (0.08/0.22)	<0.001
- day six after vaccination	0.15 (0.09/0.24)	<0.001
- day seven after vaccination	0.02 (0.01/0.05)	<0.001
Age (per 10 years)	0.86 (0.74/1.01)	0.06
Sex:		
- female	reference	
- male	0.57 (0.40/0.82)	0.002
- non-binary	0.56 (0.06/5.12)	0.61
Living arrangement:		
- living together with others	reference	
- living alone	0.97 (0.67/1.40)	0.87
Educational level (pursuant to CASMIN):		
- higher or lower tertiary education	reference	
- secondary school certificate or “A” level equivalent	1.21 (0.86/1.70)	0.29
- inadequately completed, general elementary or basic vocational	1.59 (0.71/3.54)	0.26
Migration status:		
- participant and both parents born in Germany	reference	
- participant born in Germany and at least one parent born abroad	1.43 (0.91/2.24)	0.12
- participant born abroad	1.78 (1.12/2.83)	0.02

OR: odds ratio; CI: confidence interval; n: number of participants (n = 1678); N: number of observations (N = 10 447); **CASMIN: Comparative Analysis of Social Mobility in Industrial Nations**

© 2023 Schäfer I et al. *JAMA Network Open*.

eTable 17. Associations With Deep Leg Pain: Results of Mixed-Effects Ordered Logistic Regression Analysis Adjusted for Random Effects on the Patient Level [continued]

Characteristic	OR (95% CI)	p
Self-reported health problems:		
- Back pain	1.67 (1.13/2.46)	0.009
- Depression	1.19 (0.73/1.93)	0.48
- Gastrointestinal complaints	1.94 (1.18/3.18)	0.009
- Hypertension	0.84 (0.41/1.70)	0.62
- Pulmonary disease	1.19 (0.60/2.34)	0.62
- Rheumatism or other autoimmune disease	1.06 (0.49/2.28)	0.88
- Heart disease	2.55 (1.03/6.32)	0.04
- Osteoarthritis	0.68 (0.25/1.85)	0.45
- Renal disease	0.80 (0.25/2.52)	0.70
- Anaemia or other blood disease	0.45 (0.15/1.40)	0.17
- Diabetes mellitus	0.46 (0.12/1.75)	0.25
- Cancer	0.50 (0.09/2.74)	0.43
- Liver disease	1.29 (0.23/7.10)	0.77
Anxiety or depression (pursuant to PHQ-4, per 2 points difference)	1.20 (1.02/1.41)	0.03
Vaccine:		
- BNT162b2	reference	
- mRNA-1273	2.73 (1.89/3.96)	<0.001
Somatosensory Amplification (per 8 points difference)	1.19 (0.93/1.53)	0.16
Expected benefit of vaccination (per 3 points difference)	0.74 (0.57/0.96)	<0.001
Expected risk for COVID-19 infection (per 3 points difference)	0.95 (0.77/1.18)	0.65
Expected risk for hospitalisation due to COVID-19 infection (per 3 points difference)	0.89 (0.70/1.14)	0.36
Expected risk for adverse effects of vaccination (per 3 points difference)	0.95 (0.74/1.20)	0.65
Expected risk for hospitalisation due to adverse effects of vaccination (per 3 points difference)	0.95 (0.68/1.30)	0.73
Expected risk for long-term adverse effects of vaccination (per 3 points difference)	1.19 (0.90/1.56)	0.22
Adverse effects experienced at first vaccination (per 3 points difference)	1.60 (1.27/2.01)	<0.001
Adverse effects observed in close contacts (per 3 points difference)	1.13 (0.91/1.41)	0.28

OR: odds ratio; CI: confidence interval; n: number of participants (n = 1678); N: number of observations (N = 10 447); PHQ: patient health questionnaire

© 2023 Schäfer I et al. *JAMA Network Open*.

eBox 1. Symptom Diary—English Version, Translated From German

As part of our study "Well-being after COVID-19 vaccinations", we would like to invite you to report your state of health and possible symptoms in a digital diary every evening for the first seven days after your COVID-19 vaccination.

<u>Please always relate your answers to the current day.</u>

<u>Note on fever measurement:</u> Please measure your body temperature with a standard household thermometer. Please always measure in the same place, i.e. mouth, ear or armpit.

Fever

- ☐ no fever or temperature below 37.5°C
- ☐ 37.5°C to 37.9°C
- ☐ 38.0°C to 38.4°C
- ☐ 38.5°C to 38.9°C
- ☐ 39.0°C to 40.0°C
- ☐ more than 40.0°C

Chills

- ☐ no chills
- ☐ light chills, not limiting
- ☐ moderate chills, limiting normal activities
- ☐ severe chills, stopping normal activities
- ☐ very severe chills, stopping (almost) all activities

Headache

- ☐ no headache
- ☐ light headache, not limiting
- ☐ moderate headache, limiting normal activities
- ☐ severe headache, stopping normal activities
- ☐ very severe headache, stopping (almost) all activities

© 2023 Schäfer I et al. *JAMA Network Open*.

eBox 1. Symptom diary – English version, Translated from German [continued]

Tiredness/fatigue

- ☐ no tiredness or fatigue
- ☐ light tiredness or fatigue, not limiting
- ☐ moderate tiredness or fatigue, limiting normal activities
- ☐ severe tiredness or fatigue stopping normal activities
- ☐ very severe tiredness or fatigue, stopping (almost) all activities

Joint pain

- ☐ no joint pain
- ☐ light joint pain, not limiting
- ☐ moderate joint pain, limiting normal activities
- ☐ severe joint pain, stopping normal activities
- ☐ very severe joint pain, stopping (almost) all activities

Aching limbs

- ☐ no aching limbs
- ☐ lightly aching limbs, not limiting
- ☐ moderately aching limbs, limiting normal activities
- ☐ severely aching limbs, stopping normal activities
- ☐ very severely aching limbs, stopping (almost) all activities

Vomiting

- ☐ no vomiting
- ☐ one or two times vomiting in 24 hours
- ☐ more than two times vomiting in 24 hours
- ☐ fluid substitution required due to vomiting
- ☐ fluid substitution and medical clarification required

Diarrhoea

- ☐ no diarrhoea or less than two times soft stool in 24 hours
- ☐ two or three times soft stool in 24 hours
- ☐ four or five times soft stool in 24 hours
- ☐ six or more times soft stool in 24 hours
- ☐ six or more times soft stool in 24 hours, medical clarification required

© 2023 Schäfer I et al. *JAMA Network Open*.

eBox 1. Symptom diary – English version, Translated from German [continued]

Haematomas/punctiform haemorrhages

- ☐ no haematomas or punctiform haemorrhages
- ☐ ne to five haematomas or punctiform haemorrhages
- ☐ six to ten haematomas or punctiform haemorrhages
- ☐ more than ten haematomas or punctiform haemorrhages
- ☐ more than ten haematomas or punctiform haemorrhages, medical clarification required

Deep leg pain

- ☐ no deep leg pain
- ☐ light deep leg pain, not limiting
- ☐ moderate deep leg pain, limiting normal activities
- ☐ severe deep leg pain, stopping normal activities
- ☐ very severe deep leg pain, stopping (almost) all activities

Heart pain

- ☐ no heart pain
- ☐ light heart pain, not limiting
- ☐ moderate heart pain, limiting normal activities
- ☐ severe heart pain, stopping normal activities
- ☐ very severe heart pain, stopping (almost) all activities

Shortness of breath

- ☐ no shortness of breath
- ☐ light shortness of breath, not limiting
- ☐ moderate shortness of breath, limiting normal activities
- ☐ severe shortness of breath, stopping normal activities
- ☐ very severe shortness of breath, stopping (almost) all activities

© 2023 Schäfer I et al. *JAMA Network Open*.

eBox 2. Symptom Diary—German Version

Im Rahmen unserer Studie: „Wohlbefinden nach COVID-19 Impfungen“ möchten wir Sie einladen, Ihr Befinden und mögliche Beschwerden jeden Abend in den ersten sieben Tagen nach Ihrer COVID-19 Impfung in einem digitalen Tagebuch anzugeben.

Bitte beziehen Sie Ihre Antworten immer auf den aktuellen Tag.

Hinweis zur Fiebermessung: Bitte messen Sie Ihre Körpertemperatur mit einem haushaltsüblichen Fieberthermometer. Messen Sie bitte immer an derselben Stelle, also Mund, Ohr oder Achselhöhle.

Fieber

- ☐ kein Fieber oder Temperatur unter 38,0°C
- ☐ 37,5°C to 37,9°C
- ☐ 38,0°C to 38,4°C
- ☐ 38,5°C to 38,9°C
- ☐ 39,0°C to 40,0°C
- ☐ über 40,0°C

Frösteln/Schüttelfrost

- ☐ kein Frösteln/Schüttelfrost
- ☐ mildes Frösteln/Schüttelfrost, nicht beeinträchtigend
- ☐ moderates Frösteln/Schüttelfrost, beeinträchtigt normale Aktivitäten
- ☐ starkes Frösteln/Schüttelfrost, verhindert normale Aktivitäten
- ☐ sehr starkes Frösteln/Schüttelfrost, verhindert (fast) alle Aktivitäten

Kopfschmerzen

- ☐ keine Kopfschmerzen
- ☐ milde Kopfschmerzen, nicht beeinträchtigend
- ☐ moderate Kopfschmerzen, beeinträchtigen normale Aktivitäten
- ☐ starke Kopfschmerzen, verhindern normale Aktivitäten
- ☐ sehr starke Kopfschmerzen, verhindern (fast) alle Aktivitäten

© 2023 Schäfer I et al. *JAMA Network Open*.

eBox 2. Symptom diary – German version [continued]

Müdigkeit/Abgeschlagenheit

- ☐ keine Müdigkeit/Abgeschlagenheit
- ☐ milde Müdigkeit/Abgeschlagenheit, nicht beeinträchtigend
- ☐ moderate Müdigkeit/Abgeschlagenheit, beeinträchtigt normale Aktivitäten
- ☐ starke Müdigkeit/Abgeschlagenheit, verhindert normale Aktivitäten
- ☐ sehr starke Müdigkeit/Abgeschlagenheit, verhindert (fast) alle Aktivitäten

Gelenkschmerzen

- ☐ keine Gelenkschmerzen
- ☐ milde Gelenkschmerzen, nicht beeinträchtigend
- ☐ moderate Gelenkschmerzen, beeinträchtigen normale Aktivitäten
- ☐ starke Gelenkschmerzen, verhindern normale Aktivitäten
- ☐ sehr starke Gelenkschmerzen, verhindern (fast) alle Aktivitäten

Gliederschmerzen

- ☐ keine Gliederschmerzen
- ☐ milde Gliederschmerzen, nicht beeinträchtigend
- ☐ moderate Gliederschmerzen, beeinträchtigen normale Aktivitäten
- ☐ starke Gliederschmerzen, verhindern normale Aktivitäten
- ☐ sehr starke Gliederschmerzen, verhindern (fast) alle Aktivitäten

Erbrechen

- ☐ kein Erbrechen
- ☐ ein- bis zweimal Erbrechen in 24 Stunden
- ☐ mehr als zweimal Erbrechen in 24 Stunden
- ☐ Flüssigkeitssubstitution (Infusion) aufgrund des Erbrechens erforderlich
- ☐ Flüssigkeitssubstitution (Infusion) erforderlich, ärztliche Abklärung nötig

Durchfall

- ☐ kein Durchfall oder weniger als zwei weiche Stühle in 24 Stunden
- ☐ zwei bis drei weiche Stühle in 24 Stunden
- ☐ vier bis fünf weiche Stühle in 24 Stunden
- ☐ sechs oder mehr weiche Stühle in 24 Stunden
- ☐ mehr als sechs weiche Stühle in 24 Stunden, ärztliche Abklärung nötig

© 2023 Schäfer I et al. *JAMA Network Open*.

eBox 2. Symptom diary – German version [continued]

Blaue Flecke/punktförmige Einblutungen

- ☐ keine blauen Flecke/punktförmigen Einblutungen
- ☐ ein bis fünf blaue Flecke/punktförmige Einblutungen
- ☐ sechs bis zehn blaue Flecke/punktförmige Einblutungen
- ☐ mehr als zehn blaue Flecke/punktförmige Einblutungen
- ☐ mehr als zehn blaue Flecke/punktförmige Einblutungen, ärztliche Abklärung nötig

Tiefe Beinschmerzen

- ☐ keine tiefen Beinschmerzen
- ☐ milde tiefe Beinschmerzen, nicht beeinträchtigend
- ☐ moderate tiefe Beinschmerzen, beeinträchtigen normale Aktivitäten
- ☐ starke tiefe Beinschmerzen, verhindern normale Aktivitäten
- ☐ sehr starke tiefe Beinschmerzen, verhindern (fast) alle Aktivitäten

Herzbeschwerden

- ☐ keine Herzbeschwerden
- ☐ leichte Herzbeschwerden, nicht beeinträchtigend
- ☐ moderate Herzbeschwerden, beeinträchtigen normale Aktivitäten
- ☐ starke Herzbeschwerden, verhindern normale Aktivitäten
- ☐ sehr starke Herzbeschwerden, verhindern (fast) alle Aktivitäten

Atemnot

- ☐ keine Atemnot
- ☐ milde Atemnot, nicht beeinträchtigend
- ☐ moderate Atemnot, beeinträchtigt normale Aktivitäten
- ☐ starke Atemnot, verhindert normale Aktivitäten
- ☐ sehr starke Atemnot, verhindert (fast) alle Aktivitäten

© 2023 Schäfer I et al. *JAMA Network Open*.
