# Supplementary material for: Expectations and Prior Experiences Associated With Adverse Effects of COVID-19 Vaccination
Source: JAMA Netw Open. 2023 Mar 27;6(3):e234732. doi: 10.1001/jamanetworkopen.2023.4732 (PMC10043751; doi:10.1001/jamanetworkopen.2023.4732)
Supplement: Supplement 2. — Data Sharing Statement [file jamanetwopen-e234732-s002.pdf]

Data Sharing Statement

Schäfer. Expectations and Prior Experiences Associated With Adverse Effects of COVID-19 Vaccination. *JAMA Netw Open*. Published March 27, 2023.
doi:10.1001/jamanetworkopen.2023.4732

Data
Data available: No

Additional Information
Explanation for why data not available: The consent statement did not specify that data would be published, and for this reason we are unable to deposit the data set in a public repository. However, de-identified data are available upon reasonable request from the corresponding author.
